# Multi-omic characterization of the sow colostrum and milk microbiome and proteome

**DOI:** 10.1099/mgen.0.001726

**Published:** 2026-06-17

**Authors:** Devin B. Holman, Katherine E. Gzyl, Arun Kommadath, Pekka Määttänen

**Affiliations:** 1 Lacombe Research and Development Centre, Agriculture and Agri-Food Canada, 6000 C&E Trail, Lacombe, AB, T4L1W1, Canada; 2 Department of Biology, Burman University, Chan Shun Science Centre, 6730 University Drive, Lacombe, AB, T4L 2E5, Canada

**Keywords:** antimicrobial resistance, colostrum, culturomics, metagenomics, microbiome, proteomics, sow milk

## Abstract

Sow colostrum and milk provide essential nutrients, immune protection and one of the earliest microbial exposures for piglets. However, the microbial composition, functional potential and host interactions of these mammary secretions remain poorly characterized. Here, we combined culturomics, metagenomics and proteomics to comprehensively characterize the microbiome and proteome of sow colostrum and milk collected at farrowing and at 7 and 21 days postpartum. We recovered 132 bacterial isolates representing at least 42 species, including 15 putatively novel taxa. These isolates included both potentially pathogenic species, such as *Sarcina perfringens* and *Streptococcus suis,* and potentially beneficial bacterial species like *Lactobacillus amylovorus* and *Lactiplantibacillus plantarum*. The microbial composition and functional potential shifted significantly as the milk matured, with *L. amylovorus*, *Limosilactobacillus reuteri* and *Rothia spp*. among the most relatively abundant taxa. Several antimicrobial resistance genes, including *erm*(C), *tet(K*), *tet*(M), *lnu(A*), *poxtA* and *fexB*, were identified on contigs encoding plasmid replicons in the isolates, indicating potential for horizontal gene transfer. Functional annotation of isolate genomes indicated broad carbohydrate-active enzyme (CAZyme) repertoires, including β-galactosidase-associated families and other CAZyme families consistent with potential milk oligosaccharide utilization. The colostrum and milk proteome also shifted during lactation, reflecting declining immune-related proteins and increasing metabolic and structural proteins. Correlations between specific microbial taxa and host proteins, including *Rothia spp*. and immune proteins or glycoproteins, suggested potential host–microbe interactions during lactation. Together, these findings provide a multi-omic perspective on how mammary microbiome dynamics and host responses during lactation may influence neonatal microbial colonization and health.

**Impact Statement**

Sow colostrum and milk are important early microbial exposures for piglets, yet their microbiomes remain poorly characterized. Using culturomics, metagenomics and proteomics, we provide a high-resolution analysis of these microbiomes and proteomes during lactation. The genomes of 132 bacterial isolates from these samples were sequenced and characterized, including isolates representing putatively novel taxa and 18 high-quality metagenome-assembled genomes were also recovered. The microbiomes, resistomes and proteomes all shifted significantly during the 21-day lactation period. Several plasmid-associated antimicrobial resistance genes (ARGs), including clinically relevant ARGs, such as *poxtA*, were identified in a number of the isolates, suggesting that sow colostrum and milk may be an important reservoir of antimicrobial resistance. Overall, this study expands current understanding of the functional and ecological dynamics of the sow mammary microbiome and provides a foundation for future work on host-microbe interactions during lactation, colostrum management and the design of colostrum substitutes.

## Data Summary

The isolate genomes and metagenome-assembled genomes are publicly available in the National Center for Biotechnology Information’s (NCBI) Sequence Read Archive and genome databases under BioProject PRJNA1134706. Piglet faecal and sow colostrum and milk metagenomic sequences are available under BioProject PRJNA779404. Detailed accession numbers for metagenomic and genomic data are provided in Tables S1 and S2, available in the online version of this article.

## Introduction

Sow colostrum and milk serve as the primary sources of nutrition for piglets prior to weaning. Colostrum is the initial secretion from the mammary gland, typically produced within the first 24 h of farrowing, providing piglets with energy, as well as passive immunity in the form of immunoglobulins (IgA, IgG and IgM) [1]. As mammary secretions transition to mature milk, the protein content decreases and the concentrations of lipids and lactose increase [2]. In addition to immunoglobulins, colostrum and milk contain a variety of bioactive proteins, including antimicrobial peptides, such as lactoferrin, along with enzymes and signalling factors that can directly influence microbial colonization and immune system maturation [3]. Microorganisms are also present in colostrum and milk, and are among the earliest microbial exposures for piglets [4,7].

The origin of these colostrum- and milk-associated microbes in sows and other mammals remains a subject of debate. One proposed mechanism is oro/entero-mammary translocation, whereby microbes from the gastrointestinal tract or oral cavity are transported via immune cells to the mammary gland [8]. It is also possible that microbes on the skin and in the oral cavity of the offspring may enter the mammary gland (retrograde transfer) [8]. Regardless of their origin, colostrum and milk are important sources of early-life microbial exposure for piglets that shape the neonatal gut microbiome, modulate immune system development and influence growth and health outcomes [9]. Therefore, a clearer understanding of the composition and functional potential of the sow colostrum and milk microbiome is needed to evaluate how these microbes may contribute to piglet health and dissemination of antimicrobial resistance.

Previous studies using 16S rRNA gene sequencing have shown that the composition of the sow colostrum and milk microbiota changes over the lactation period [5] and includes members of the *Clostridium*, *Corynebacterium*, *Lactobacillus*, *Streptococcus* and *Staphylococcus* genera [10,11]. However, these studies are limited by the inherent constraints of 16S rRNA gene sequencing with respect to both functional and taxonomic resolution. As a result, the broader community structure, functional potential and relationships between the milk microbiome and host-derived proteins remain incompletely understood. Higher-resolution metagenomic sequencing can mitigate some of these limitations by enabling species- and strain-level identification together with functional gene profiling. Integrating microbiome and proteomic data can further improve understanding of host-microbe interactions in the mammary gland. In the present study, we used both metagenomic sequencing and culturomics to profile sow colostrum and milk at the community, functional, species and strain levels, and integrated these data with proteomic analyses of the same samples. We hypothesized that sow colostrum and milk would contain microbial communities with distinct functional potential over lactation, including potentially beneficial bacteria as well as other taxa that may serve as reservoirs of antimicrobial resistance genes (ARGs), and that the proteome would include host proteins associated with immune function and microbial colonization.

## Methods

### Animals and experimental design

Pigs were cared for in accordance with the guidelines of the Canadian Council on Animal Care. All procedures and protocols involving animals were reviewed and approved by the Lacombe Research and Development Centre Animal Care Committee (animal use protocol number 201905). Oxytocin was administered to Landrace × Yorkshire sows (*n*=14) inseminated with Duroc semen to ensure farrowing within the same 24 h period. Colostrum (day of farrowing) and milk samples (7 and 21 days post-farrowing) were collected into sterile 150 ml screw-cap containers using sterile gloves, following thorough cleaning of the teat with 0.5% hydrogen peroxide (Prevail disinfectant wipes, Virox Technologies Inc., Oakville, ON, Canada). Samples were stored on ice and transported to the laboratory within 3 h of collection. The sows were housed in individual farrowing stalls and did not receive any antimicrobials during gestation or lactation.

### Bacterial isolation

Colostrum and milk samples were processed for culturing in a vinyl anaerobic chamber (Coy Laboratory Products, Grass Lake, MI, USA) supplied with a premixed gas of 10% H_2_, 10% CO_2_ and 80% N_2_. Samples were diluted tenfold in PBS (Thermo Scientific, Mississauga, ON, Canada) containing 0.1 µM resazurin sodium salt (MilliporeSigma, Oakville, ON, Canada) and 8 mM sodium sulfite. From this dilution, 100 µl was spread onto agar plates with de Man, Rogosa and Sharpe (Thermo Scientific), Wilkins-Chalgren Anaerobe (Thermo Scientific), Tryptone Soya (TS) (Thermo Scientific) or TS supplemented with 5% sterile rumen fluid (Bar Diamond, Parma, ID, USA). Plates were incubated anaerobically at 39 °C for 48 h using an AnaeroGen system (Thermo Scientific), corresponding to the body temperature of sows. At least one colony was selected from each agar type based on morphology using a sterile toothpick and transferred to a screw-cap tube containing 350 µl of the corresponding broth with 15% glycerol. The tube caps were loosened and the tubes were incubated anaerobically at 39 °C for 48 h. Caps were then tightened and cultures stored at −80 °C until reculturing.

### Bacterial isolate DNA extraction

Bacterial isolates were regrown on their original agar with the exception that TS with 5% rumen fluid was replaced with TS agar. Isolates were recultured under the same conditions used for the initial isolation. A single colony was then selected and inoculated into a Hungate tube (Chemglass Life Sciences, Vineland, NJ, USA) containing 10 ml of the corresponding broth for 16 h at 39 °C. A DNeasy Blood and Tissue kit (Qiagen, Mississauga, ON, Canada) was used to extract genomic DNA from each culture according to the manufacturer’s protocol.

### Initial bacterial isolate screening

A total of 271 isolates were recovered and screened via Sanger sequencing of the near-full-length 16S rRNA gene. The 16S rRNA gene was amplified using the primers 8F (5′-AGA GTT TGA TCC TGG CTC AG-3′) and 1492R (5′-CGG TTA CCT TGT TAC GAC TT-3′) [12] together with a HotStarTaq Plus Master Mix kit (Qiagen). Briefly, in a total reaction volume of 20 µl, 0.2 µM of each primer was included along with 1X CoralLoad Concentrate, 2 ng of template DNA and 1X HotStarTaq Plus Master Mix in molecular-grade water. The PCR conditions consisted of an initial 95 °C heat activation step for 5 min, followed by 35 cycles of denaturation at 94 °C for 1 min, annealing at 55 °C for 1 min and extension at 72 °C for 2 min, with a final extension at 72 °C for 10 min. The PCR amplicons (20 µl) were electrophoresed on a 0.8% agarose gel (Invitrogen, Waltham, MA, USA) for 45 min at 100 V. A single ~1,500 bp band was excised from the gel and purified with a QIAquick gel extraction kit (Qiagen) as per the manufacturer’s instructions. Each purified PCR product was sent to Eurofins (Louisville, KY, USA) for Sanger sequencing with the 16S F standard primer option. The 16S rRNA gene sequences were then identified using BLASTn [13].

### Whole-genome sequencing of bacterial isolates

Based on the 16S rRNA gene classification, isolates representing lactic acid-producing species, potentially pathogenic species such as *Streptococcus suis*, potentially novel species and at least one representative of each additional species identified were selected for whole-genome sequencing. In addition to the isolates screened via Sanger sequencing of the 16S rRNA gene, 53 isolates were included without this screening step, resulting in a total of 132 isolates subjected to whole-genome sequencing. Genomic libraries were prepared using an Illumina DNA Prep Library kit as previously described [14], and sequenced on an Illumina MiSeq instrument (v3 reagent kit: 600 cycles; v2 reagent kit: 300 cycles).

Long-read sequencing was also conducted on isolates representing potentially novel bacterial species and on at least one strain from species within the genera *Lactiplantibacillus*, *Lactobacillus*, *Latilactobacillus*, *Limosilactobacillus* and *Weissella*. For PacBio HiFi sequencing, DNA was extracted with the Nanobind CBB kit (Pacific Biosciences, Menlo Park, CA, USA) following the HMW DNA extraction protocol for gram-positive bacteria provided by the manufacturer. To enhance cell lysis efficiency, cell pellets were resuspended in 10 µl of PBS containing 10 mg ml^−1^ MetaPolyzyme (MilliporeSigma) and incubated for 30 min at 35 °C, followed by a 90 min incubation with 10 mg ml^−1^ lysozyme in STET buffer (8% sucrose, 50 mM Tris-HCl, 50 mM EDTA, 5% Triton X-100). Genomic DNA from these isolates was purified using a DNeasy PowerClean Pro CleanUp kit (Qiagen) and sequencing libraries were prepared with the SMRTbell Prep kit 3.0 (Pacific Biosciences) following the manufacturer’s instructions.

Briefly, purified DNA (final volume: 65 µl) was sheared using a Megaruptor 3 instrument (Diagenode Inc., Denville, NJ, USA). Sequencing primer 3.2 was annealed and the Sequel II 3.2 polymerase was bound. Libraries underwent AMPure bead cleanup following the SMRT Link v. 11.0 calculator procedure before sequencing on a PacBio Sequel II instrument at a loading concentration of 90 pM, using the adaptive loading protocol with the Sequel II Sequencing kit 2.0, SMRT Cell 8M and 15 h movies with a 2 h pre-extension time. For isolates sequenced on the MinION R10.4.1 flow cell (Oxford Nanopore, Oxford, UK), DNA extraction, library preparation and sequencing were carried out as described previously [15].

### Bacterial genome assembly and analysis

Illumina raw reads were processed, quality-filtered and assembled as described in Holman *et al*. [15] using the same parameters and software versions. Similarly, the processing and assembly of Nanopore reads were performed as previously detailed [15]. Processing and assembly of the PacBio reads were similar to those used for the Nanopore reads with the exception that the minimum read length was set to 1,000 bp in Filtlong v.0.2.1 (https://github.com/rrwick/Filtlong) and Medaka polishing was omitted in Trycycler v.0.5.4 [16]. Assemblies were identical before and after polishing, except for *Lactobacillus amylovorus* 11266D007BMRS-1, for which the short-read-polished assembly was retained. For all genome assemblies, taxonomy was assigned using the Genome Taxonomy Database toolkit (GTDB-Tk) v.2.4.1 and the GTDB release 10-R226 [17]. CheckM2 v.1.0.1 [18] was used to assess completeness and contamination and QUAST v.5.2.0 [19] was used to determine assembly metrics. ARGs were identified with the resistance gene identifier (RGI) v.6.0.3 and the Comprehensive Antibiotic Resistance Database (CARD) v.4.0.0 [20], retaining strict and perfect hits only and using AMRFinderPlus v.4.2.7 (database version 2026-01-21.1) [21]. Only ARGs identified by both tools were retained for downstream analysis. Genome assemblies were screened for copper and zinc resistance genes using BacMet v.2.0 [22] and for virulence-associated genes using the virulence factor database (February 13, 2026 release) [23]. Protein-coding genes were predicted with Prodigal v.2.6.3 [24] and aligned against these databases using DIAMOND BLASTP v.2.1.23 [25] with an e-value threshold of 1×10⁻¹⁰, minimum sequence identity of 80% and minimum query and subject coverage of 80%. Plasmid replicons were identified with PlasmidFinder v.2.1.6–1 [26]. The average nucleotide identity (ANI) between isolates was calculated using fastANI v.1.33 [27]. For isolates identified as *Escherichia coli*, serotype was determined *in silico* using SerotypeFinder v.2.0 [28].

### Metagenomic DNA extraction, sequencing and analysis

Details of DNA extraction from colostrum and milk samples, metagenomic library preparation and initial sequence processing are available in Holman *et al*. [15]. Kraken2 v.2.1.6 [29] and Bracken v.3.0.1 [30] with the GTDB release 10-R226 were used to classify the unassembled sequences. The Kraken2- and Bracken-formatted GTDB also included the pig (Sscrofa11.1) and human (GRCh38.p14) genomes. ARGs were identified with RGI v.6.0.3 and CARD v.4.0.0 with KMA. The metagenomes were assembled both individually for each sample and as a co-assembly using MEGAHIT v.1.2.9 [31]. Reads were then mapped back to each respective individual assembly as well as the co-assembly using Bowtie2 v.2.5.1 [32] and the contigs were binned into metagenome-assembled genomes (MAGs) with MetaBAT 2 v.2 : 2.15 [33]. The completeness and contamination of the MAGs were assessed with CheckM v.1.2.2 and those MAGs with >90% completeness and <5% contamination were retained. Redundant MAGs were removed with dRep v.3.4.3 [34] with primary and secondary clustering at 90 and 99% ANI, respectively. A phylogenomic tree of the MAGs and isolate genomes was constructed using PhyloPhlAn v.3.1.68 [35] and visualized in iTOL v.6.9.1 [36].

The unassembled metagenomic reads were aligned against the prokaryotic protein database from Kyoto Encyclopedia of Genes and Genomes (KEGG) release 112.0 [37] using DIAMOND BLASTx. Gene hits were assigned to KEGG orthologs (KOs) and KO abundances were normalized by sequencing depth. KOs were then filtered with MinPath [38] to identify a parsimonious set of pathways, and the remaining KOs were assigned to KEGG pathways. The relative abundances of MAGs and isolates in sow milk and colostrum samples were estimated using CoverM v.0.7.0 [39]. Reads from untreated and pre-weaned piglets from these sows were mapped to these MAGs and isolate genomes as described in Holman *et al*. [15]. Both the isolate genomes and MAGs were analysed using run_dbcan v.5.2.1 with DIAMOND and PyHMMER v.0.11.0 [40] under default settings against the dbCAN3 database (release: 2025-09-13) [41] to annotate carbohydrate-active enzymes (CAZymes) and identify CAZyme gene clusters (CGCs) and their predicted polysaccharide substrates.

### Proteomics

The protein content of the colostrum and milk was assessed at the Proteomics Core Facility, Université de Montréal (Montreal, QC, Canada). An initial acetone precipitation was performed on 40 µl of each colostrum and milk sample. The samples were then centrifuged and the supernatant was removed. The pellet was reconstituted in 100 mM Tris-HCl (pH 8.2) containing 8 M urea, vortexed and centrifuged at 17,000×***g*** for 10 min. Supernatants were collected and protein concentrations were determined using the Bradford assay. The solution containing 100 µg of protein was diluted in 100 mM Tris-HCl with 1 M urea and 10 mM TCEP [Tris(2-carboxyethyl)phosphine hydrochloride; Thermo Scientific], and vortexed for 1 h at 37 °C. Chloroacetamide (Sigma-Aldrich) was then added to a final concentration of 40 mM for alkylation and the samples were vortexed for an additional hour at 37 °C. One microgram of trypsin was then added, and digestion was performed for 8 h at 37 °C. The samples were next desalted on ultra-microspin columns (The Nest Group Inc., Ipswich, MA, USA), dried and solubilized in 5% acetonitrile and 4% formic acid. The samples were then loaded onto a 1.5 µl pre-column (Optimize Technologies, Oregon City, OR, USA).

Peptides were separated on a homemade reversed-phase column (150 µm inner diameter×200 mm) with a 56 min gradient from 10–30% acetonitrile and 0.2% formic acid and a 600 nl min^−1^ flow rate on an Easy-nLC 1200 system coupled to a Q Exactive HF Orbitrap LC-MS/MS System (Thermo Scientific). Each full MS spectrum, acquired at a resolution of 120,000, was followed by acquisition of tandem mass spectra on the most abundant multiply charged precursor ions for 3 s. Tandem MS experiments were performed using higher-energy collision dissociation at a collision energy of 34%. The data were processed using PEAKS X Pro (Bioinformatics Solutions, Waterloo, ON) and the UniProt *S. scrofa* database. Mass tolerances on precursor and fragment ions were 10 ppm and 0.01 Da, respectively. Carbamidomethylation was used as a fixed modification. Variable post-translational modifications included acetylation, oxidation, deamidation and phosphorylation. The data were visualized with Scaffold 5.0 using a protein threshold of 99%, with at least two peptides identified and a peptide-level false discovery rate (FDR) of 1%.

### Statistical analysis

All statistical analyses were conducted in R v.4.5.1. The composition of the microbiome (species relative abundances), resistome (ARGs; counts per million reads) and KOs (counts per million reads) were assessed across sampling days using Bray–Curtis dissimilarities and permutational multivariate analysis of variance (PERMANOVA), implemented with adonis2 in vegan v.2.7-1 [42]. For each analysis, sampling day was included as the explanatory variable and permutations were constrained within sow ID to account for repeated longitudinal sampling of the same animals. Pairwise PERMANOVA comparisons between day 0 and day 7, day 0 and day 21 and day 7 and day 21 were conducted using the same permutation constraint. MaAsLin3 v.1.1.0 [43] was used to identify differentially abundant ARGs, microbial species and KEGG pathways in pairwise comparisons between sampling days. For each analysis, sampling day was included as a fixed effect and sow as a random effect to account for repeated longitudinal sampling of the same animals. Models were fitted using log-transformed abundances, using a minimum prevalence threshold of 25%, and significance was assessed using the Benjamini–Hochberg (BH) procedure (FDR<0.05). Inverse Simpson diversity was also calculated for microbial species, ARGs and KOs. All analyses used mixed-effects models with day as a fixed effect and sow as a random effect. The species inverse Simpson diversity values were log-transformed prior to analysis because the raw data were non-normal overall. Pairwise comparisons among days were adjusted using the BH procedure.

The protein abundance table exported from Scaffold 5.0, which reports relative intensities, was analysed after removing proteins deleted from UniProt and matching gene names to their corresponding UniProt entries. Principal component analysis (PCA) was carried out using PCAtools v.2.16.0 [44], after excluding the 10% least variable proteins. Scores for the first and second principal components, together with the top five contributing proteins for each component, were extracted and visualized with ggplot2 v.3.5.2 [45]. Gene names were dereplicated by retaining the first occurrence, and loading arrows were scaled to match the range of PCA scores.

Protein abundance values were normalized with cumulative sum scaling in the metagenomeSeq package v.1.46.0 [46] and then log_2_-transformed. Differential abundance analysis was performed with limma v.3.60.6 [47] by fitting a linear model across all samples with time included as a factor. A contrast matrix was constructed to compare day 0 vs. day 7, day 0 vs. day 21 and day 7 vs. day 21. Moderated t-statistics and *P*-values were obtained using empirical Bayes and *P*-values were adjusted with the BH procedure. Volcano plots were generated with thresholds of absolute log_2_ fold change greater than one and adjusted *P*-values below 0.05. For each comparison, the five most significant proteins were labelled with their UniProt gene names.

Correlations between the microbiome (species) and proteome were evaluated in vegan via Procrustes analysis based on Bray–Curtis dissimilarity non-metric multidimensional scaling (NMDS) ordinations. In addition, pairwise Spearman rank correlations were calculated between microbial species abundance values and protein abundance values across all samples. Prior to correlation analysis, the protein dataset was reduced to the 500 proteins with the highest variance across samples, and microbial species were filtered to retain those with non-zero values in more than 20% of samples. The *P*-values were adjusted for multiple comparisons with the BH procedure.

Gene ontology (GO) biological process enrichment analysis was performed using the PANTHER GO Enrichment Analysis tool v.19.0 [48] with default settings and the *S. scrofa* reference database. For each comparison, the input consisted of the differentially abundant proteins (UniProt entries) with a log₂ fold change greater than 0.5 and an FDR of less than 0.05. Enriched GO terms were then mapped to higher-level summary categories using the R packages ontologyIndex v.2.12, GO.db v.3.19.1, AnnotationDbi v.1.66.0 and the GOslim generic ontology database (v. 22 July 2025).

## Results

### Bacteria isolated from sow colostrum and milk

**Fig. 1.**
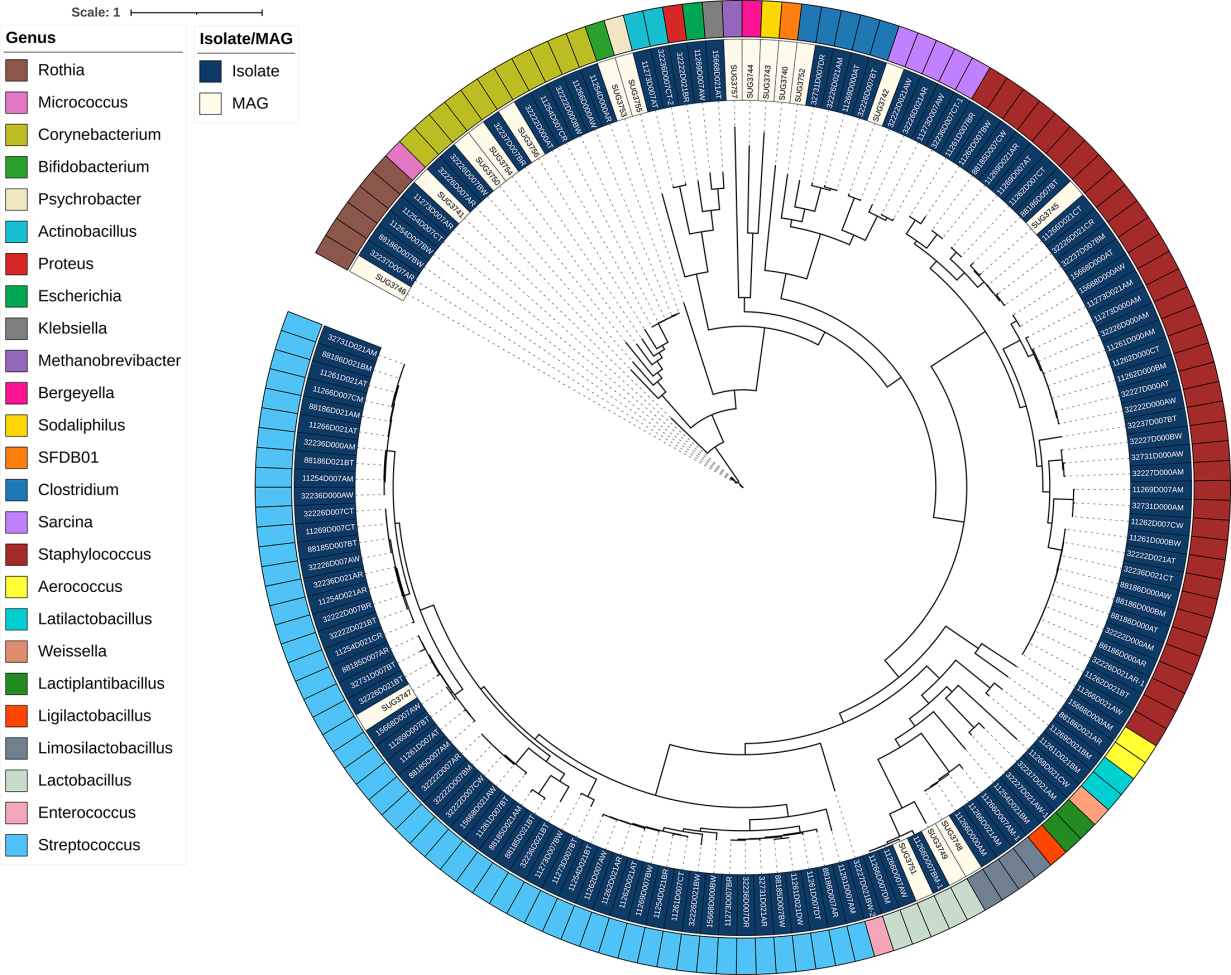
Maximum-likelihood phylogenetic tree of metagenome-assembled genomes (MAGs) (inner ring, white) and isolate genomes (inner ring, blue). Genus-level classifications are indicated by outer ring colours and the legend follows the clockwise order of genera around the tree.

The 132 isolates whose genomes were sequenced and classified belonged to at least 42 unique species within three phyla: Actinobacteriota, Bacillota and Pseudomonadota (Fig. 1 and Table S2). Fifteen isolates were identified as putatively novel species, based on GTDB-Tk classification and further evaluation using the Type Strain Genome Server (TYGS) [49], or as members of species-level taxa lacking cultured representatives. These isolates belonged to the *Corynebacterium*, *Rothia*, *Staphylococcus* and *Streptococcus* genera. The majority of isolates (*n*=91) were classified as either *Staphylococcus* or *Streptococcus spp*., representing at least 19 different species.

One of the objectives of this study was to isolate *Lactobacillaceae* bacteria, as members of this family have been well established to have beneficial properties in various animal hosts, including pigs. Twelve *Lactobacillaceae* isolates were recovered and identified as *Lactiplantibacillus plantarum*, *L. amylovorus*, *Latilactobacillus curvatus*, *Ligilactobacillus salivarius*, *Limosilactobacillus reuteri* or *Weissella paramesenteroides*. Potentially pathogenic or zoonotic strains were also recovered from colostrum and milk, including *Aerococcus suis*, *E. coli*, *S. suis* and *Sarcina* (*Clostridium*) *perfringens. In silico* serotyping identified the *E. coli* isolate as serotype O6:H7. Using a≥99.99% ANI to define strains as recommended by Rodriguez-R *et al*. [50], there were potentially 12 unique *S. suis* strains represented among the isolate collection.

### Antimicrobial resistance genes in isolates

**Fig. 2.**
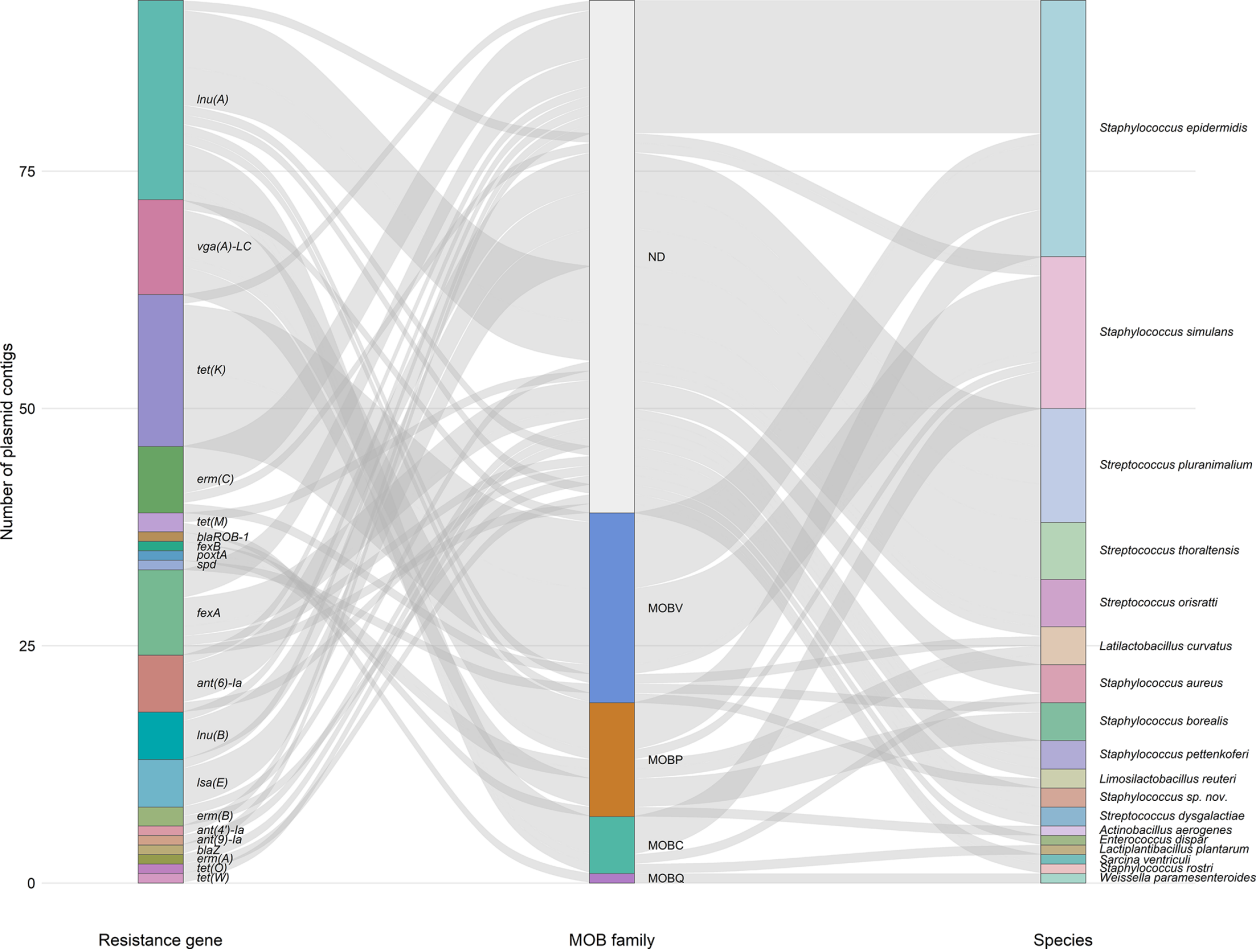
Alluvial plot showing the distribution of antimicrobial resistance genes (ARGs) across plasmid mobility (MOB) families and host species. Flow width represents the number of plasmid contigs carrying each ARG. ARGs (left) are connected to MOB families (centre) and bacterial species (right). MOB families are classified based on relaxase typing, with ‘ND’ indicating no MOB relaxase detected.

The most frequently detected ARGs were *ant(6)-Ia*, *erm*(B), *lnu*(A), *lnu*(B), *lsa*(E) and *tet*(O) (>20 isolates). With the exception of one isolate (11254D021BT), all *S. suis* isolates (*n*=14) carried at least one tetracycline resistance gene, including *tet*(40), *tet*(45), *tet*(L), *tet*(M), *tet*(O) or *tet*(O/W/32/O). Overall, 54 isolate assemblies contained ARGs located on contigs predicted to be plasmid-derived (Fig. 2 and Table S3). Notably, *L. curvatus* 11269D021BM carried two distinct ARG-encoding plasmids: a rep29 plasmid (p11269D021BM-1; 25,787 bp) harbouring *fexB* and *poxtA* and a rep28 plasmid (p11269D021BM-2; 10,359 bp) encoding *tet*(M) (Table S3). For plasmid p11269D021BM-1, the closest match in the NCBI Nucleotide database was an *Enterococcus faecium* plasmid (99.71% identity, 88% coverage; accession CP072887.1) from an isolate recovered from surface water [51].

The p11269D021BM-1 plasmid also encoded a MOBP relaxase, suggesting it may be mobilizable, provided a functional origin of transfer and conjugation machinery are available. Plasmid p11269D021BM-2 aligned most closely with a *L. plantarum* plasmid (99.87% identity, 87% coverage; accession: AF440277.1) isolated from corn silage [52]. In addition to phenicols, the *poxtA* gene confers resistance to oxazolidinones [53], which include linezolid, an antibiotic of last resort used to treat serious infections caused by gram-positive bacteria, such as methicillin-resistant *Staphylococcus aureus* (MRSA) and vancomycin-resistant enterococci (VRE) [54]. *Weissella paramesenteroides* 11269D021CW also carried a larger plasmid (16,510 bp) harbouring *tet*(M). Although no known replicon type was identified, the plasmid encoded a MOBQ relaxase, indicating it may be mobilizable.

Other ARGs located on contigs predicted to be plasmid-derived included *tet*(K) (rep7a) in several *Staphylococcus epidermidis* and *Staphylococcus simulans* isolates, as well as *vga*(A)_LC_ (rep5b, rep5d, rep10b) in many of the same *S. epidermidis* isolates (Table S3). The *tet*(K)-encoding plasmid contigs ranged from 4,517 to 4,630 bp in length and were predicted to be mobilizable based on the presence of a MOBV relaxase. Similarly, the *vga*(A)_LC_-encoding plasmid contigs were predicted to be mobilizable, as they carried a MOBP relaxase gene. The *lnu*(A) gene conferring resistance to lincosamides was associated with three different replicon types depending on the species and strain: rep13 (*S. simulans*), rep33 (*Streptococcus orisratti*, *Streptococcus thoraltensis*) and repUS54 (*Enterococcus dispar* 32227D021BWCA-2 and *L. curvatus* 11261D021BM). The aminoglycoside resistance gene *ant(4′)-Ia* was co-located with rep22 in *S. epidermidis* 11262D021BT, although no MOB gene was detected.

The isolate genomes were also screened for copper and zinc resistance genes, as these metals have historically been used at supranutritional concentrations in swine production, potentially contributing to the co-selection and persistence of antimicrobial resistance. The three *S. aureus* genomes all carried a multicopper oxidase (*mco*) gene, as did 20 additional *Staphylococcus* isolates (Table S2). Copper resistance genes were detected in *Aerococcus urinaeequi* (*tcrA*, *tcrB*, *tcrZ*) and *E. dispar (tcrB* and *tcrZ*) isolates, while *tcrB* alone was identified in 39 isolates. Multiple genes associated with zinc resistance (*zitB*, *zntA*, *zntR*, *zraR* and *zraS*) were only detected in *E. coli*, which also encoded copper resistance genes (*copA*, *cueO*, *cueR*, *cusA*, *cusB*, *cusC, cusF/cusX, cusR* and *cusS*). None of the copper or zinc resistance genes were co-located with an ARG on a contig classified as being of plasmid origin.

### Virulence factor detection in isolates

Among all isolates evaluated for virulence-associated genes*, E. coli* 11269D007AW, classified *in silico* as serotype O6:H7, had the most extensive virulence-associated gene profile. This included multiple genes encoding adhesins associated with type 1, P, S and F1C fimbriae, as well as *fdeC* (Table S4). Additional genes were associated with alpha-hemolysin (*hlyABCD*), cytotoxic necrotizing factor 1 (*cnf1*), a partial colibactin biosynthetic gene cluster, multiple iron acquisition systems and group 2 capsule biosynthesis. The isolate also encoded *tcpC* and the invasion-associated genes *ibeB* and *ibeC. Klebsiella pneumoniae* 15668D021AT also had multiple virulence-associated genes, including type 1 and type 3 fimbriae and the iron acquisition systems yersiniabactin, salmochelin and aerobactin (Table S4).

The three *S. aureus* isolates (11269D007AM, 11262D007CW and 32731D000AM) had similar virulence-associated gene profiles, including genes associated with adhesion, biofilm formation, capsule type 8 biosynthesis, iron-regulated surface determinants, hemolysins and immune evasion (Table S4); isolate 11269D007AM additionally encoded *spa* (staphylococcal protein A). Among the remaining isolates with virulence-associated genes identified, the three *S. perfringens* isolates had the largest number of virulence-associated genes, including alpha-toxin (*cpa*/*plc*) and, in two isolates, perfringolysin O (*pfoA*), together with multiple exoenzyme genes. Isolate *S. perfringens* 32236D007CT-1 also encoded the beta2-toxin gene, *cpb2*.

### Carbohydrate-active enzymes in isolates

The isolate genomes were analysed for CAZymes, CGCs and their predicted polysaccharide substrates to examine their distribution and provide insight into the functional potential of the isolates. CAZymes catalyse the breakdown, modification and synthesis of carbohydrates, while CGCs comprise physically linked genes encoding at least one CAZyme, a transporter, a transcriptional regulator and a signal transduction protein involved in carbohydrate metabolism [55]. Glycoside hydrolases (GHs), which hydrolyse glycosidic bonds, are the largest class of CAZymes. The isolates with the greatest number of unique GH families were those classified as *S. perfringens, Clostridium nitritogenes, K. pneumoniae, E. coli* and *S. suis* (Table S2). The most frequently encoded GHs were GH13 (98.5% of 132 genomes), GH32 (93.9%), GH1 (88.6%), GH73 (87.9%) and GH23 (84.8%). Another CAZyme class, the glycosyltransferases (GTs), which are involved in glycan synthesis, was also widespread; GT2, GT4, GT28, GT51 and GT119 were identified in all isolate genomes.

**Fig. 3.**
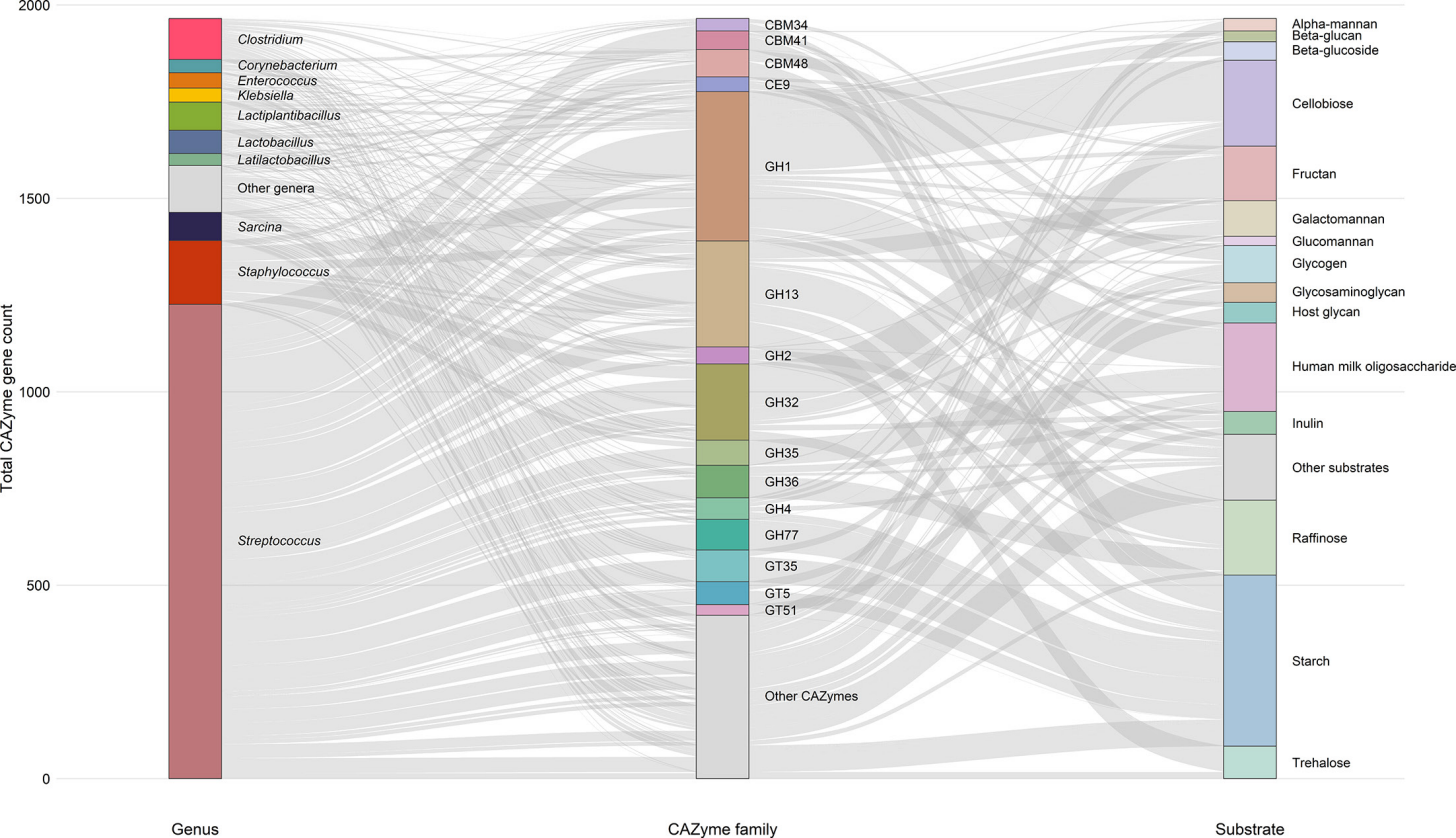
Alluvial diagram showing the distribution of CAZyme families across bacterial genera isolated from colostrum and milk samples. Flows represent CAZyme gene counts linking genus, CAZyme family and predicted substrate. Analysis was restricted to the ten most abundant genera and the fifteen most abundant CAZyme families and substrates; remaining categories were grouped as ‘Other’. Stratum height and flow width are proportional to gene count.

A total of 32 polysaccharides were predicted to serve as substrates for the detected CGCs. Starch, human milk oligosaccharides, fructan, trehalose, galactomannan and raffinose were among the most common substrates, each associated with CGCs in at least 60 genomes (Fig. 3). Nearly all genomes (93.9%) encoded at least one GH family (GH1, GH2, GH35 or GH42) with β-galactosidase activity, conferring the ability to hydrolyse lactose, the most abundant carbohydrate in sow colostrum and milk [3]. *Corynebacterium* sp028726465, *Corynebacterium* sp024580955 and *Rothia* sp034179845 were the only isolates lacking all of these GH families. In addition to GH2 and GH42, other GH families involved in the breakdown of milk oligosaccharides include GH20 (β-hexosaminidases), GH29 and GH95 (fucosidases), GH33 (sialidases), GH112 (galacto-N-biose/lacto-N-biose phosphorylases) and GH136 (lacto-N-biosidases) [56]. With the exception of GH136, the *S. perfringens* isolates encoded CAZymes from all of these families, indicating their ability to metabolize milk oligosaccharides.

### Sow colostrum and milk metagenomes

As reported previously, the vast majority of sequences in the colostrum and milk metagenomes originated from the host (97.1±1.2%, sem) [15]. The average number of reads per sample after quality-filtering and host sequence removal was 2,018,105±896,045 (data not shown). Metagenomic reads from the colostrum and milk samples, as well as from faeces collected from the piglets, were aligned to the isolate genomes to assess their relative abundance. For the colostrum, the *L. amylovorus* and *L. reuteri* genomes as well as two *Rothia spp*. genomes (88186D007BWCA and 32237D007ATSR) were the only isolates with a relative abundance greater than 0.1% (Table S2). In the milk collected on days 7 and 21, all unidentified *Rothia spp*. isolates were relatively abundant (>0.1%), along with isolates classified as *A. urinaeequi*, *L. amylovorus*, *L. reuteri*, *Sarcina ventriculi* and *Staphylococcus hyicus*. The *E. coli* isolate was by far the most abundant in the piglet faecal microbiome (11.7±0.8%) followed by *S. perfringens* and the *L. amylovorus* isolates (≥1.0%).

### Colostrum and milk microbiome composition, resistome and functional potential

When the colostrum and milk metagenomic reads were profiled against the GTDB, the most relatively abundant species across all samples were *Methanocatella* sp900769095 (NCBI: *Methanobrevibacter sp*.), *L. amylovorus*, *Rothia* sp034090985, *Rothia* sp034179845 and *S. suis* (Table S5). It is important to note that 24.1±3.9% of the reads were assigned to the pig genome despite a host read removal step during pre-processing (data not shown). This highlights the importance of including host genomes in the Kraken2 database for taxonomic classification of low-biomass samples and is consistent with the findings of Gihawi *et al*. [57].

**Fig. 4.**
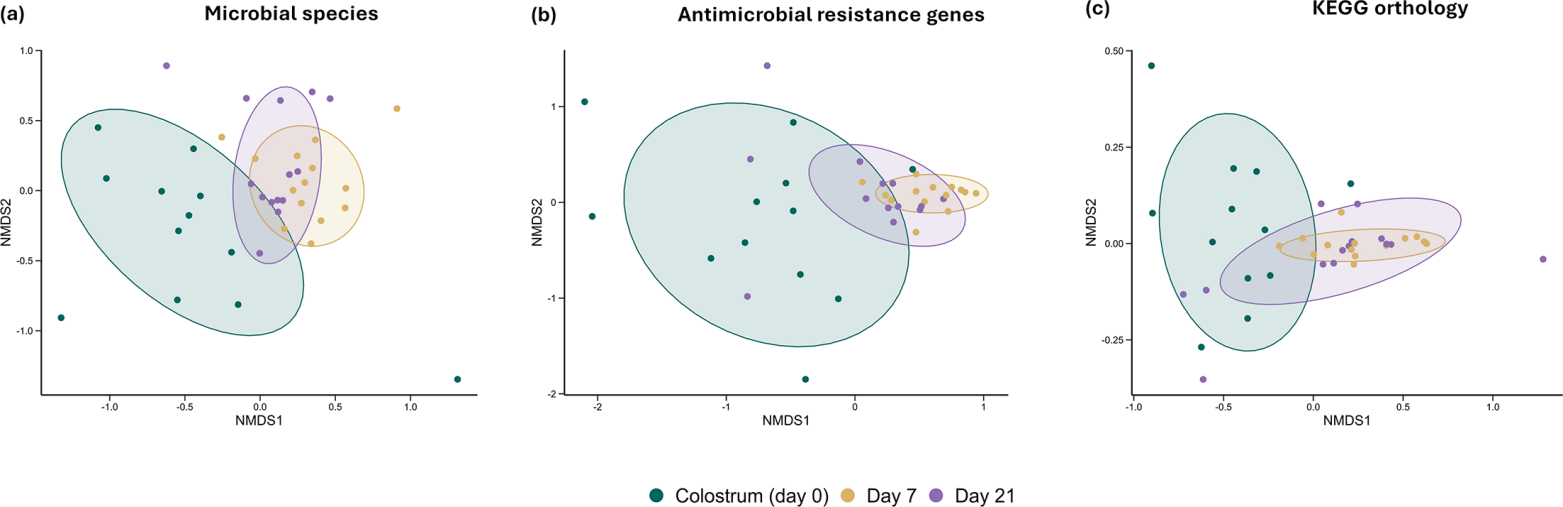
Non-metric multidimensional (NMDS) ordinations of day 0 (colostrum), day 7 and day 21 samples based on Bray–Curtis dissimilarities of (**a**) archaeal and bacterial species relative abundances, (**b**) antimicrobial resistance gene abundances (counts per million reads) and (**c**) KO relative abundances (counts per million reads). Points represent individual samples and ellipses denote 80% confidence intervals around group centroids.

The microbial species composition of the colostrum and milk microbiomes differed significantly across all sampling time points (PERMANOVA: *R²* = 0.21, *P*<0.001; Fig. 4a). The most pronounced shifts were observed between day 0 (colostrum) and day 7 (*R²* = 0.19, *P*<0.001), as well as between day 0 and day 21 (*R²* = 0.18, *P*<0.001). Significant differences were also detected between day 7 and day 21 (*R²* = 0.11, *P*<0.001). Microbial species diversity (inverse Simpson diversity) was also significantly lower in the day 0 samples compared to days 7 and 21 (*P*<0.05; Fig. S1a). A total of 41 species were identified as differentially abundant between day 0 and day 7, while 32 species differed between day 0 and day 21 (FDR<0.05; Table S6). Of these, 18 species were consistently differentially abundant in both the day 0 vs. day 7 and day 0 vs. day 21 comparisons. The majority of these species were more abundant in the day 0 colostrum samples. Species enriched in the colostrum samples included *Aeromonas salmonicida*, *Lelliottia chinensis*, *Serratia proteamaculans* and *Streptococcus faecavium*, whereas *Prevotella* sp002251245, *Rothia* sp034090985, *S. suis* (GTDB species cluster V) and *Turicibacter bilis* were more abundant in the day 7 and 21 milk samples. Additionally, 57 bacterial species were differentially abundant between the day 7 and 21 samples.

Distinct shifts in resistome composition were also observed over the lactation period (PERMANOVA: *R²* = 0.17, *P*<0.001; Fig. 4b), with the most substantial changes occurring between day 0 and day 7 (*R²* = 0.18, *P*<0.001) and between day 0 and day 21 (*R²* = 0.12, *P*<0.001). ARG diversity was significantly lower in day 0 samples than in day 7 and day 21 samples, and day 7 samples had greater ARG diversity than day 21 samples (*P*<0.05; Fig. S1b). Despite these significant shifts in overall composition and diversity, relatively few individual ARGs were differentially abundant between day 0 and either day 7 or day 21. Specifically, *lnu*(C), *lsa*(E) and *tet*(M) were enriched in day 7 samples, while *tet*(40), *tet*(44) and *tet*(O/W) were enriched in day 21 samples compared to day 0 (FDR<0.05). The most abundant ARGs across all samples were those conferring resistance to tetracyclines, such as *tet*(W), *tet*(Q), *tet*(K) and *tet*(O) and to the macrolides-lincosamides-streptogramin B class, including *lnu*(A) and *lnu*(C) (Table S7). Consistent with the isolate data, several of the ARGs most frequently detected in the isolate collection, including *tet*(O), *lnu*(A), *lnu*(C), *ant(6)-Ia*, *erm*(B), *lnu*(B) and *lsa*(E), were also among the 50 most abundant ARGs in the milk metagenomes.

Similar to the species and ARG composition, the functional composition of the milk microbiome based on KO abundances also differed significantly between day 0 and day 7 (*R^2^*=0.18, *P*=0.002) and between day 0 and day 21 (*R^2^*=0.11, *P*=0.006) (Fig. 4c). KO diversity was also significantly greater in day 7 and day 21 samples than in day 0 samples (*P*<0.05; Fig. S1c). KOs were further assigned to KEGG pathways and compared across sampling times. There were 41 functional pathways enriched in day 7 relative to day 0 and 38 were enriched in day 21 relative to day 0 (FDR<0.05). Steroid degradation was the only pathway that was more abundant in the day 0 (colostrum) samples than in later milk samples, and this difference was observed only in the comparison with day 21. There were no differentially abundant KEGG pathways between the day 7 and day 21 samples (FDR>0.05; Table S8). Pathways related to carbohydrate metabolism (e.g. phosphotransferase system, starch and sucrose metabolism, galactose metabolism), lipid metabolism (glycerophospholipid metabolism, biosynthesis of unsaturated fatty acids) and energy production (oxidative phosphorylation, nitrogen metabolism, porphyrin metabolism) were among those significantly more abundant in the microbiomes of day 7 and day 21 milk.

### Metagenome-assembled genomes

The metagenomic reads were assembled and binned into 18 high-quality MAGs (≥90% completeness; ≤5% contamination) (Fig. 1 and Table S9). Six of these MAGs corresponded to species also recovered in the isolate collection, including *L. amylovorus*, *L. reuteri*, *S. ventriculi*, *Rothia* sp034179845, *S. chromogenes* and *Streptococcus hyovaginalis*. In the phylogenetic tree, all of these MAGs clustered with at least one isolate genome. For example, the *S. ventriculi* MAG (SUG3742) grouped with the S. *ventriculi* isolate (32222D021AW), although the *lnu*(P) and *tet*(O) genes identified in the isolate were not binned into the MAG (Fig. 1 and Table S9). Likewise, *L. amylovorus* isolates 11266D007AW and 11266D007DM and MAG SUG3751 were highly similar, as were *S. hyovaginalis* isolates 32226D021BT, 32731D007BT, 88185D007AR and MAG SUG3747.

Species recovered only as MAGs included *Bergeyella zoohelcum*, *Bifidobacterium mongoliense*, *Psychrobacter sanguinis* and the archaeal placeholder *Methanocatella* sp900769095. The MAGs classified as *L. amylovorus*, *Rothia* sp034179845 and *L. reuteri* were most relatively abundant in both the colostrum and milk samples (Table S9). Within piglet faecal microbiomes, the *L. amylovorus, Lactobacillus* sp910589675*, L. reuteri* and *Sodaliphilus* sp004557565 MAGs were most relatively abundant (>0.1%). Although many ARGs detected in isolates were not binned into MAGs, *ant(6)-Ia, aph(3′)-Ia,* and *tet*(W/N/W) were detected in MAGs classified as *L. amylovorus, P. sanguinis* and *L. reuteri*, respectively.

### Sow colostrum and milk proteomes

**Fig. 5.**
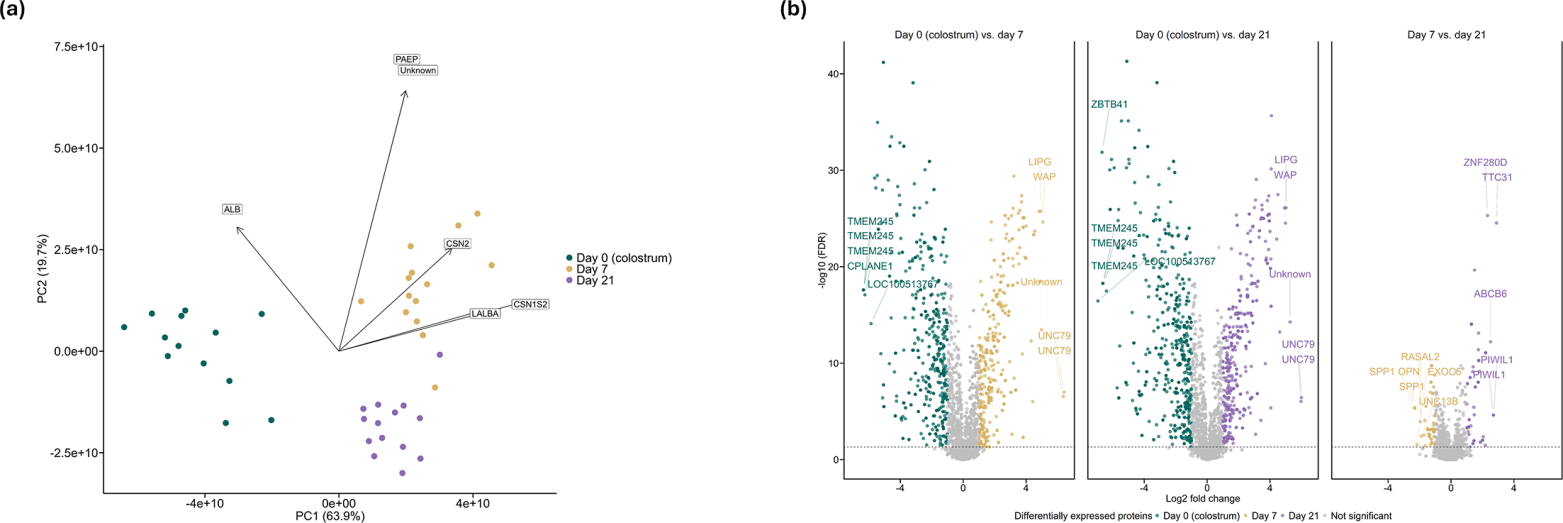
(**a**) PCA biplot of colostrum (day 0) (*n*=13), day 7 (*n*=14) and day 21 (*n*=14) samples based on proteomic profiles. Arrows indicate protein loadings that contribute most strongly to separation along the first two principal components. (**b**) Volcano plots of differentially abundant proteins for pairwise comparisons (colostrum vs. day 7, colostrum vs. day 21 and day 7 vs. day 21). Coloured points denote proteins enriched in each group, grey points indicate non-significant changes and selected significant proteins are labelled. The horizontal dashed line marks the FDR threshold for significance.

The proteomes of the colostrum and milk were analysed to assess changes in protein composition during lactation and their potential relationship with the microbiome. Caseins and whey proteins, such as albumin (ALB), α-lactalbumin (LALBA), β-lactoglobulin and whey acidic protein, along with immune-related proteins, were relatively abundant across colostrum and milk samples (Table S10). As with the microbiomes, the proteomes of the colostrum and milk samples also differed significantly by time (Fig. 5a). Of the 3,680 proteins quantified, colostrum (day 0) and mature milk (day 21) exhibited the greatest divergence, with 681 proteins significantly more abundant in the colostrum and 523 proteins significantly more abundant in the mature milk (FDR<0.05) (Figs 5b, S2 and Table S11). Similarly, the transition from colostrum to early milk (day 0 vs. day 7) was marked by substantial differences, with 569 proteins significantly more abundant in day 0 samples and 461 in day 7 samples (FDR<0.05). In contrast, relatively few differences were detected between early and mature milk (day 7 vs. day 21), with only 59 proteins significantly more abundant in the day 7 milk samples and 63 in the day 21 samples (FDR<0.05).

**Fig. 6.**
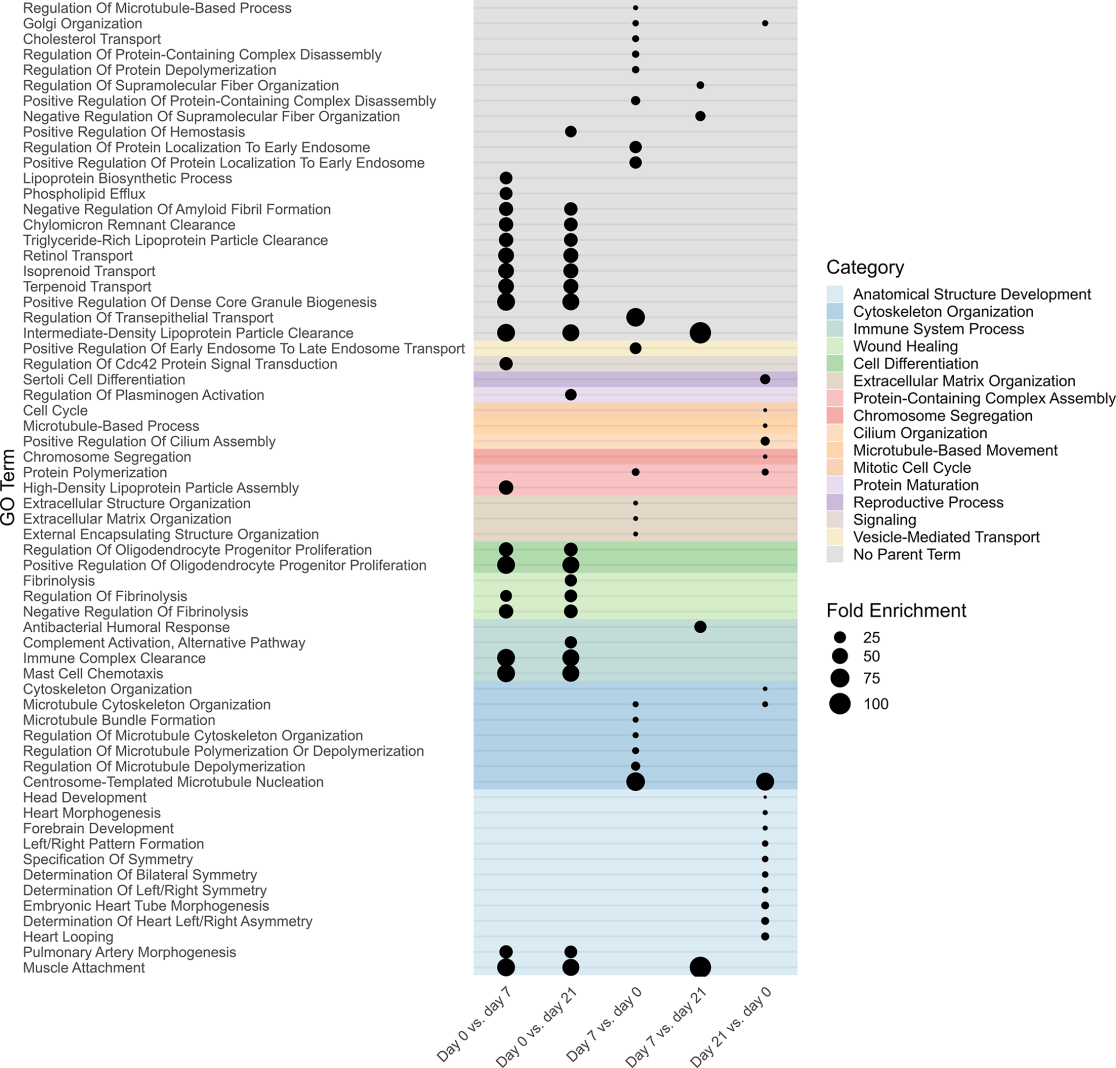
The 20 most significantly enriched GO biological processes identified in sow colostrum and milk within each pairwise time-point comparison. Dot size represents fold enrichment, while colours indicate functional categories of the GO terms.

Several of the whey proteins were among those differentially abundant between colostrum and mature milk (day 7 and 21) samples, with ALB, serotransferrin (TF) and haptoglobin enriched in the colostrum and LALBA and lactoperoxidase (LPO) enriched in the day 7 and 21 samples (Table S11 and Fig. S2). Among the proteins most strongly linked with colostrum samples were colostrum trypsin inhibitor-like protein, ciliogenesis and planar polarity effector complex subunit 1 and several zinc finger proteins. The differentially abundant proteins were assigned to GO terms for functional annotation. Among the most enriched GO terms in colostrum were those related to immune system processes and response, as well as wound healing and cell differentiation (Fig. 6 and Table S12). Many of the GO terms enriched among the day 7 and 21 samples compared to day 0 were those associated with cytoskeleton organization.

**Fig. 7.**
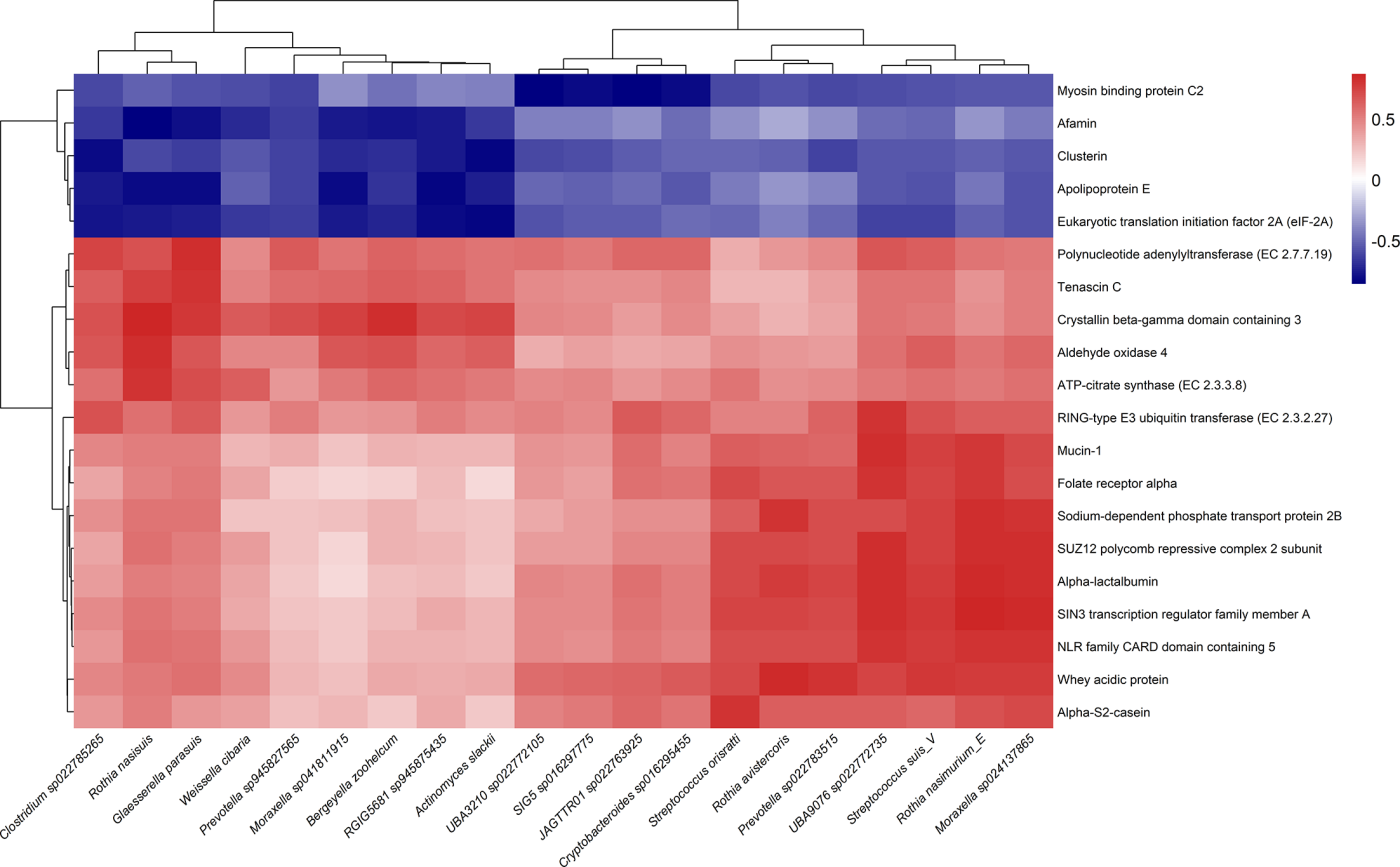
Heatmap of Spearman correlation coefficients between the 20 proteins and 20 bacterial species most frequently represented among the top 1,000 ranked protein–species correlations identified across all samples (FDR<0.05).

A significant but weak correlation was observed between the microbiome and proteome of the colostrum and milk samples (Procrustes correlation=0.29; *P*=0.029). Correlations between individual species and proteins were also calculated. The strongest associations included *Rothia nasisuis* with crystallin beta-gamma domain containing 3 (Spearman’s *r*=0.87) and afamin (*r*=−0.85) and *Rothia nasimurium* with SIN3 transcription regulator family member A (*r*=0.86) and LALBA (r=0.85) (Fig. 7 and Table S13). Other notable positive correlations were observed between mucin-1 and *Moraxella* sp024137865, *R. nasimurium*, *S. suis* (V) and UBA9076 sp022772735. The relative abundance of the swine pathogen *Glaesserella parasuis* correlated with several proteins, including polynucleotide adenylyltransferase (*r*=0.83), apolipoprotein E (APOE) (*r*=−0.82), granulin (*r*=−0.81), tenascin C (*r*=0.81) and afamin (*r*=−0.80).

## Discussion

In addition to serving as a primary source of nutrients and immunoglobulins, sow colostrum and milk represent one of the earliest microbial exposures for the piglet gastrointestinal tract. Although previous studies have isolated bacteria from sow colostrum and milk [6] and profiled the colostrum and milk microbiota [5,10, 11], their reliance on 16S rRNA gene sequencing for microbial identification and characterization is limiting. To address this knowledge gap, we characterized the microbiome of sow colostrum and milk using both culturomics and metagenomic sequencing. In parallel, we analysed the host-derived proteomes of these samples and investigated potential associations between the milk microbiome and host proteome.

We recovered 132 bacterial isolates representing at least 42 unique species, including 15 putatively novel species. The collection included several potentially beneficial bacteria, such as *L. amylovorus*, *L. curvatus, L. salivarius, L. plantarum* and *L. reuteri*. Among these, the *L. amylovorus* isolates were the most relatively abundant in the colostrum and milk metagenomes and were also relatively abundant in piglet faecal microbiomes during the nursing period, suggesting potential maternal transfer. Certain *L. amylovorus* strains have been shown to reduce adhesion of enterotoxigenic *E. coli* (ETEC) and mitigate epithelial cell damage *in vitro* [58,59] and to lower the abundance of ETEC in the ileum of pigs *in vivo* when delivered via feed in challenge experiments [60]. Similarly, *L. reuteri*, which was also relatively abundant in colostrum and milk, has been linked through dietary supplementation to increased average daily gain and reduced diarrhoeal incidence in pigs [61]. As such, it is clear that sow colostrum and milk serve as an early source of beneficial lactobacilli that contribute to piglet health and development.

While sow colostrum and milk contain beneficial bacteria, such as lactobacilli, they may also be a source of pathogenic or opportunistic species, including isolates identified as *A. suis*, *S. hyicus*, *S*. (*Clostridium*) *perfringens* and *S. suis*. The only publicly available genome of *A. suis* beyond our isolate is that of the type strain, originally obtained from the brain of a pig with meningitis [62]. *S. hyicus* is the main etiologic agent of exudative epidermitis, also known as ‘greasy pig disease’ [63]. *S. suis* is a leading cause of meningitis and sepsis [64] and *S. perfringens* can cause necrotic enteritis in piglets, depending on toxinotype [65]. However, these species are also part of the commensal microbiome in swine, and because the isolates were recovered from sow milk and colostrum, their detection should be interpreted cautiously, as they may reflect transient contamination during sampling rather than true intramammary colonization. Consistent with this interpretation, *S. perfringens* was relatively abundant in the piglet gastrointestinal tract (>0.5%), yet all piglets remained healthy.

However, several isolates encoded known virulence-associated genes. The recovered *E. coli* strain carried multiple virulence-associated genes associated with extraintestinal pathogenic *E. coli*, including *papC*, *sfa*/*foc*-associated genes, *kps* capsule genes, *hlyA* and *cnf1* [66]. Strains from the same *E. coli* serotype (O6:H7) have previously been recovered from swine faeces [67]. Similarly, the *S. perfringens* isolates carried several toxin- and exoenzyme-associated genes, including *cpa*/*plc*, *pfoA*, *colA*, sialidase genes, hyaluronidase genes and *cloSI*, which are recognized components of the *S. perfringens* virulence repertoire [68]. One *S. perfringens* isolate also carried the beta2-toxin gene, *cpb2*, which has been linked to porcine enteric disease, although its contribution to intestinal pathogenicity is not completely resolved [69].

Antimicrobial resistance remains a serious challenge in both human and veterinary medicine. Sow colostrum and milk may represent a potential route for the transfer of antimicrobial-resistant bacteria to piglets. Therefore, in the present study, we screened the isolates for known ARGs, focusing on those associated with mobile genetic elements, such as plasmids. As expected, the most frequently detected ARGs were those corresponding to antimicrobials historically used in swine production, including aminoglycosides, macrolides and lincosamides and tetracyclines. Several ARGs were identified on the same contig as plasmid replicons. Among these was *tet*(K), a tetracycline efflux pump gene co-located with rep7a in a number of *S. epidermidis* and *S. simulans* isolates. The *tet*(K) gene has previously been found with rep7a in multiple *Staphylococcus* species, including *S. epidermidis* from pigs and swine farmers [70].

One *L. curvatus* isolate (11269D021BM) carried *fexB* and *poxtA* together on a plasmid (rep29) and *tet*(M) on a separate plasmid (rep28). The rep29 plasmid was predicted to be mobilizable based on the presence of a MOBP relaxase, although it would require conjugative elements available in trans to be transferred. Both *fexB* and *poxtA* confer resistance to florfenicol, which is used in swine production to prevent and treat bacterial infections. However, *poxtA* also mediates resistance to the oxazolidinones [71], a class of antimicrobials not approved for use in veterinary medicine because they are considered medically important for humans. The oxazolidinones include linezolid, a drug often reserved for treating serious infections, such as MRSA and VRE [54,72]. Recently, we also identified *fexB* and *poxtA* co-located on a plasmid in florfenicol- and linezolid-resistant *Enterococcus avium*, *Enterococcus faecalis* and *E. faecium* isolates recovered from piglets treated with florfenicol [15]. Plasmid-encoded *fexB* and *poxtA* have also been detected in a *L. salivarius* strain isolated from a pig [73]. These findings emphasize the need for careful screening of lactobacilli strains for ARGs during probiotic development.

In addition to the cultured isolates, we assembled MAGs from the colostrum and milk metagenomes. These included species, such as *B. zoohelcum* and *Sodaliphilus* sp004557565 from the phylum Bacteroidota, as well as *B. mongoliense* from the phylum Actinomycetota; none were recovered through culture-based methods. This likely reflects limitations in the media and culturing conditions used, as no members of the Bacteroidota were isolated. Although seemingly rare, *B. zoohelcum* was recently associated with respiratory disease in pigs [74], while *B. mongoliense* has been found in bovine milk and raw milk cheese with several strains able to degrade milk oligosaccharides [75].

In total, 9 of the 18 high-quality MAGs assembled represented archaeal or bacterial taxa without a cultured representative. This included an archaeal MAG classified as *Methanocatella* sp900769095 (NCBI: *Methanobrevibacter sp*.), which was relatively abundant in the colostrum and milk samples. Although *Methanobrevibacter spp*. have been isolated from human colostrum and milk [76], the biological significance of its presence in sow milk remains unclear and warrants further confirmation. Notably, one MAG (SUG3746) was assigned to the same novel species (*Rothia* sp034179845) as two isolates (32237D007AR and 88186D007BW). Several other MAGs, including *L. amylovorus* SUG3751, *S. hyovaginalis* SUG3747 and *S. ventriculi* SUG3742, were closely related to at least one isolate, demonstrating the value of MAGs in capturing both cultured and uncultured members of the colostrum and milk microbiome.

The taxonomic composition and functional potential of the sow milk microbiome shifted significantly over the course of lactation. The transition from colostrum (day 0) to early mature milk (day 7) was marked by changes in the relative abundance of a large number of archaeal and bacterial species, as well as KEGG functional pathways encoded by these microbes. *Rothia* sp034090985 was among those species with the largest relative increase in day 7 and 21 milk compared to colostrum. However, other *Rothia spp*. were relatively abundant in the colostrum, including *Rothia* sp034179845, a placeholder species from which we recovered two isolates. Other abundant *Rothia spp*. were *R. nasimurium and Rothia endophytica. Rothia spp*. are commonly reported among the abundant bacteria in sow colostrum and milk [77], as well as in the porcine tonsil [78], upper respiratory tract [79], oral cavity [11] and skin [80] microbiomes. They are present in the pig gastrointestinal tract as well, although typically at a lower relative abundance compared with other body sites [81].

Although certain *Rothia spp*., such as *R. nasimurium* isolated from pigs, have previously been reported to carry CAZymes capable of metabolizing milk oligosaccharides and mucins [82], none of the *Rothia spp*. isolate genomes in the current study encoded CAZymes specifically associated with milk oligosaccharide degradation. Milk oligosaccharides are glycans composed of a lactose core linked to monosaccharides, such as fucose and *N*-acetylglucosamine, as well as sialic acids including *N*-acetylneuraminic acid and, in pigs, *N*-glycolylneuraminic acid [83]. These oligosaccharides are notable because they are not typically digested by the piglet but are instead metabolized by specific members of the gut microbiome, thereby providing benefits to the host, such as selective stimulation of beneficial bacteria, production of short-chain fatty acids (SCFAs) and modulation of the immune system. Due to structural similarities, many of the CAZymes in the milk oligosaccharide-associated GH families may also be involved in the degradation of other host glycans, such as mucins [84]. Mucins are high-molecular-weight glycoproteins that constitute the major structural components of mucus lining the epithelial surfaces of mammals; consequently, the ability to metabolize mucins can enhance bacterial colonization of the host [85].

Among the bacterial isolates, the *S. perfringens* genomes encoded the greatest number of CAZyme families linked to milk oligosaccharide metabolism. *In vitro*, *S. perfringens* has been shown to metabolize several human milk oligosaccharides, producing SCFAs, such as butyrate and propionate [86]. Although none of the three *S. perfringens* isolate genomes were relatively abundant in the colostrum or milk, they were relatively abundant in the piglet faecal metagenomes, suggesting that the presence of these CAZymes, if expressed, may provide an early colonization advantage through the degradation of dietary milk oligosaccharides or host mucins. Lactose is the major carbohydrate in colostrum and milk, and tends to increase in concentration over the lactation period [87]. Given this, it is not surprising that nearly all isolated genomes encoded at least one GH family associated with β-galactosidase activity, enabling hydrolysis of lactose into glucose and galactose. More broadly, these CAZyme profiles may help guide future nutritional strategies that use carbohydrate substrates to preferentially support putatively beneficial bacteria associated with sow colostrum and milk. However, these interpretations are based on predicted genomic potential and will require experimental validation.

We also characterized the milk proteome and examined its association with the microbiome to better understand potential host-microbe interactions. In agreement with previous studies on sow colostrum and milk, proteins such as APOE, APOA4, APOH, ITIH2, PAPLN, TIMP2 and TF were associated with colostrum and LALBA, LPO and MUC1 with mature milk [88,89]. These proteins reflect the transition from colostral proteins involved in nutrient and lipid transport and immunity to mature milk proteins associated with antimicrobial activity and lactose synthesis. Among the most strongly colostrum-associated proteins was a colostrum trypsin inhibitor-like protein, the gene for which has been reported to be expressed over sevenfold higher in sow colostrum compared to mature milk [90]. This protein likely helps protect colostral immunoglobulins from degradation by trypsin secreted by the piglet small intestine [90]. The concentration of mucin-1, a transmembrane mucin, was strongly correlated with the relative abundance of *R. nasimurium. Rothia spp*. typically produce enzymes capable of degrading mucins [82]; however, as mentioned earlier, the *Rothia spp*. isolated in the present study lacked most CAZymes required for host glycan metabolism.

*Glaesserella parasuis*, a swine pathogen, was positively correlated with tenascin C. This extracellular matrix glycoprotein can bind to many different ligands, including toll-like receptor 4, initiating the release of pro-inflammatory cytokines. *G. parasuis* was also negatively correlated with afamin, a glycoprotein that has been found to be decreased in the serum of patients with certain infections or diseases [91]. Similarly, the relative abundance of *G. parasuis* was also negatively correlated with APOE, a lipid-binding protein. Interestingly, APOE has been shown to have antibacterial activity against certain gram-negative bacteria *in vitro* and *in vivo* in mice [92]. These correlations suggest a potential association between *G. parasuis* abundance and host inflammatory and defence proteins, consistent with a possible localized host response that may influence bacterial persistence in milk.

The exact source of bacteria present in mammalian colostrum and milk remains unclear. However, oro/entero-mammary translocation from the gastrointestinal tract or oral cavity to the mammary gland has been proposed as a plausible explanation for at least some of the species detected [8,93]. Despite careful cleaning and disinfection of the teat area prior to sample collection, certain bacterial species identified in this study likely originated from the skin surrounding the teat or from environmental sources, including faecal contamination. This is particularly relevant given that milk and colostrum represent samples with low microbial biomass, as evidenced by the high proportion of host-derived sequences (>97%) observed in the metagenomic data, which increases susceptibility to background contamination and may influence both culture-based and sequencing-based results.

In addition, metagenomic analyses were based on a relatively small proportion of non-host reads, which, although typical for milk samples, limits the depth and quantitative interpretation of microbial and functional profiles. Regardless of their precise origin, the bacteria identified in this study represent microbes that piglets naturally ingest while nursing. However, caution is warranted when interpreting these findings in terms of microbial function and host interaction, and future studies incorporating improved sampling controls and quantitative validation approaches will be important to strengthen biological inference. These early microbial exposures may nonetheless contribute to the development of the neonatal gut microbiome and immune system maturation, although their specific roles remain to be fully elucidated.

## Conclusion

Using both culturomics and metagenomic sequencing, sow milk and colostrum were found to contain a diverse microbiome that changes during lactation. Although the role of many of these bacterial species remains unclear, some, such as the lactobacilli, likely contribute to piglet health during the nursing period. Given the number of ARGs identified in the bacterial isolates, particularly those associated with plasmids, sow colostrum and milk may represent an early source of ARGs and antimicrobial-resistant bacteria for piglets. The colostrum and milk proteomes showed corresponding shifts in immune- and metabolism-related proteins across lactation, and their correlations with specific microbial taxa suggest potential host-microbe interactions during lactation. This study provides a foundation for future work investigating how colostrum and milk microbiomes influence early-life microbial colonization, and may inform strategies to enhance piglet health through microbiome manipulation.

## Supplementary material

10.1099/mgen.0.001726 Supplementary Material 1.

10.1099/mgen.0.001726 Supplementary Material 2.

## Abbreviations

ALB: albumin
APOE: apolipoprotein E
ARG: antimicrobial resistance gene
BH: Benjamini–Hochberg
CARD: Comprehensive Antibiotic Resistance Database
CAZyme: carbohydrate-active enzyme
CGC: carbohydrate-active enzyme gene cluster
cnf1: cytotoxic necrotizing factor 1
ETEC: enterotoxigenic *Escherichia coli*
FDR: false discovery rate
GH: glycoside hydrolase
GO: gene ontology
GT: glycosyltransferase
GTDB-Tk: Genome Taxonomy Database toolkit
KEGG: Kyoto Encyclopedia of Genes and Genomes
KO: Kyoto Encyclopedia of Genes and Genomes ortholog
LALBA: α-lactalbumin
LPO: lactoperoxidase
MAG: metagenome-assembled genome
MRSA: methicillin-resistant *Staphylococcus aureus*
NCBI: National Center for Biotechnology Information
NMDS: non-metric multidimensional scaling
PCA: principal component analysis
PERMANOVA: permutational multivariate analysis of variance
RGI: resistance gene identifier
SCFA: short-chain fatty acid
TF: serotransferrin
VRE: vancomycin-resistant enterococci

## References

[1] Quesnel H, Farmer C, Devillers N (2012). Colostrum intake: influence on piglet performance and factors of variation. Livest Sci.

[2] Loisel F, Farmer C, Ramaekers P, Quesnel H (2013). Effects of high fiber intake during late pregnancy on sow physiology, colostrum production, and piglet performance. J Anim Sci.

[3] Inoue R, Tsukahara T (2021). Composition and physiological functions of the porcine colostrum. Anim Sci J.

[4] Greiner LL, Humphrey DC, Holland SN, Anderson CJ, Schmitz-Esser S (2022). The validation of the existence of the entero-mammary pathway and the assessment of the differences of the pathway between first and third parity sows. *Transl Anim Sci*.

[5] Chen W, Mi J, Lv N, Gao J, Cheng J (2018). Lactation stage-dependency of the sow milk microbiota. Front Microbiol.

[6] Wang L, Liu Q, Chen Y, Zheng X, Wang C (2022). Antioxidant potential of *Pediococcus pentosaceus* strains from the sow milk bacterial collection in weaned piglets. Microbiome.

[7] Ohgi R, Saha S, Zhou B, Sakuma T, Sakurai M (2024). *In vitro* evaluation of the immunomodulatory and wakame assimilation properties of *Lactiplantibacillus plantarum* strains from swine milk. Front Microbiol.

[8] Moossavi S, Azad MB (2020). Origins of human milk microbiota: new evidence and arising questions. Gut Microbes.

[9] Nowland TL, Kirkwood RN, Pluske JR (2022). Review: can early-life establishment of the piglet intestinal microbiota influence production outcomes?. Animal.

[10] Llauradó-Calero E, Climent E, Chenoll E, Ballester M, Badiola I (2022). Influence of dietary n-3 long-chain fatty acids on microbial diversity and composition of sows’ feces, colostrum, milk, and suckling piglets’ feces. Front Microbiol.

[11] Piirainen V, König E, Husso A, Heinonen M, Iivanainen A (2025). Bacterial profiles of the oral, vaginal, and rectal mucosa and colostrum of periparturient sows. PLoS One.

[12] Galkiewicz JP, Kellogg CA (2008). Cross-kingdom amplification using bacteria-specific primers: complications for studies of coral microbial ecology. Appl Environ Microbiol.

[13] Camacho C, Coulouris G, Avagyan V, Ma N, Papadopoulos J (2009). BLAST+: architecture and applications. BMC Bioinformatics.

[14] Holman D, Agriculture and Agri-Food Canada (2023). *Salmonella* prevalence is low in deep tissue lymph nodes of hog carcasses from a pork processing plant in Alberta, Canada. FPT.

[15] Holman DB, Gzyl KE, Kommadath A (2024). Florfenicol administration in piglets co-selects for multiple antimicrobial resistance genes. mSystems.

[16] Wick RR, Judd LM, Cerdeira LT, Hawkey J, Méric G (2021). Trycycler: consensus long-read assemblies for bacterial genomes. Genome Biol.

[17] Chaumeil PA, Mussig AJ, Hugenholtz P, Parks DH (2019). GTDB-Tk: a toolkit to classify genomes with the Genome Taxonomy Database. Bioinformatics.

[18] Chklovski A, Parks DH, Woodcroft BJ, Tyson GW (2023). CheckM2: a rapid, scalable and accurate tool for assessing microbial genome quality using machine learning. Nat Methods.

[19] Gurevich A, Saveliev V, Vyahhi N, Tesler G (2013). QUAST: quality assessment tool for genome assemblies. Bioinformatics.

[20] Alcock BP, Huynh W, Chalil R, Smith KW, Raphenya AR (2023). CARD 2023: expanded curation, support for machine learning, and resistome prediction at the Comprehensive Antibiotic Resistance Database. Nucleic Acids Res.

[21] Feldgarden M, Brover V, Gonzalez-Escalona N, Frye JG, Haendiges J (2021). AMRFinderPlus and the Reference Gene Catalog facilitate examination of the genomic links among antimicrobial resistance, stress response, and virulence. Sci Rep.

[22] Pal C, Bengtsson-Palme J, Rensing C, Kristiansson E, Larsson DGJ (2014). BacMet: antibacterial biocide and metal resistance genes database. Nucl Acids Res.

[23] Zhou S, Liu B, Zheng D, Chen L, Yang J (2025). VFDB 2025: an integrated resource for exploring anti-virulence compounds. Nucleic Acids Res.

[24] Hyatt D, Chen G-L, Locascio PF, Land ML, Larimer FW (2010). Prodigal: prokaryotic gene recognition and translation initiation site identification. BMC Bioinformatics.

[25] Buchfink B, Reuter K, Drost HG (2021). Sensitive protein alignments at tree-of-life scale using DIAMOND. Nat Methods.

[26] Carattoli A, Zankari E, García-Fernández A, Voldby Larsen M, Lund O (2014). *In silico* detection and typing of plasmids using PlasmidFinder and plasmid multilocus sequence typing. Antimicrob Agents Chemother.

[27] Jain C, Rodriguez-R LM, Phillippy AM, Konstantinidis KT, Aluru S (2018). High throughput ANI analysis of 90K prokaryotic genomes reveals clear species boundaries. Nat Commun.

[28] Joensen KG, Tetzschner AMM, Iguchi A, Aarestrup FM, Scheutz F (2015). Rapid and easy *in silico* serotyping of *Escherichia coli* isolates by use of whole-genome sequencing data. J Clin Microbiol.

[29] Wood DE, Lu J, Langmead B (2019). Improved metagenomic analysis with Kraken 2. Genome Biol.

[30] Lu J, Breitwieser FP, Thielen P, Salzberg SL (2017). Bracken: estimating species abundance in metagenomics data. PeerJ Comput Sci.

[31] Li D, Liu C-M, Luo R, Sadakane K, Lam T-W (2015). MEGAHIT: an ultra-fast single-node solution for large and complex metagenomics assembly via succinct de Bruijn graph. Bioinformatics.

[32] Langmead B, Salzberg SL (2012). Fast gapped-read alignment with Bowtie 2. Nat Methods.

[33] Kang DD, Li F, Kirton E, Thomas A, Egan R (2019). MetaBAT 2: an adaptive binning algorithm for robust and efficient genome reconstruction from metagenome assemblies. PeerJ.

[34] Olm MR, Brown CT, Brooks B, Banfield JF (2017). dRep: a tool for fast and accurate genomic comparisons that enables improved genome recovery from metagenomes through de-replication. ISME J.

[35] Asnicar F, Thomas AM, Beghini F, Mengoni C, Manara S (2020). Precise phylogenetic analysis of microbial isolates and genomes from metagenomes using PhyloPhlAn 3.0. Nat Commun.

[36] Letunic I, Bork P (2024). Interactive Tree of Life (iTOL) v6: recent updates to the phylogenetic tree display and annotation tool. Nucleic Acids Res.

[37] Kanehisa M, Furumichi M, Sato Y, Matsuura Y, Ishiguro-Watanabe M (2025). KEGG: biological systems database as a model of the real world. Nucleic Acids Res.

[38] Ye Y, Doak TG (2009). A parsimony approach to biological pathway reconstruction/inference for genomes and metagenomes. PLoS Comput Biol.

[39] Aroney STN, Newell RJP, Nissen JN, Camargo AP, Tyson GW (2025). CoverM: read alignment statistics for metagenomics. Bioinformatics.

[40] Larralde M, Zeller G (2023). PyHMMER: a Python library binding to HMMER for efficient sequence analysis. *Bioinformatics*.

[41] Zheng J, Ge Q, Yan Y, Zhang X, Huang L (2023). dbCAN3: automated carbohydrate-active enzyme and substrate annotation. Nucleic Acids Res.

[42] Oksanen J, Simpson G, Blanchet F, Kindt R, Legendre P (2025). vegan: community ecology package.

[43] Nickols WA, Kuntz T, Shen J, Maharjan S, Mallick H (2026). MaAsLin 3: refining and extending generalized multivariable linear models for meta-omic association discovery. Nat Methods.

[44] Blighe K, Lun A (2025). PCAtools: everything principal components analysis.

[45] Wickham H (2016). ggplot2: Elegant Graphics for Data Analysis.

[46] Paulson JN, Stine OC, Bravo HC, Pop M (2013). Differential abundance analysis for microbial marker-gene surveys. Nat Methods.

[47] Ritchie ME, Phipson B, Wu D, Hu Y, Law CW (2015). limma powers differential expression analyses for RNA-sequencing and microarray studies. Nucleic Acids Res.

[48] Thomas PD, Ebert D, Muruganujan A, Mushayahama T, Albou L-P (2022). PANTHER: Making genome-scale phylogenetics accessible to all. Protein Sci.

[49] Freese HM, Meier-Kolthoff JP, Sardà Carbasse J, Afolayan AO, Göker M (2026). TYGS and LPSN in 2025: a Global Core Biodata Resource for genome-based classification and nomenclature of prokaryotes within DSMZ Digital Diversity. Nucleic Acids Res.

[50] Rodriguez-R LM, Conrad RE, Viver T, Feistel DJ, Lindner BG (2024). An ANI gap within bacterial species that advances the definitions of intra-species units. mBio.

[51] Biggel M, Nüesch-Inderbinen M, Jans C, Stevens MJA, Stephan R (2021). Genetic context of *optrA* and *poxtA* in florfenicol-resistant enterococci isolated from flowing surface water in Switzerland. Antimicrob Agents Chemother.

[52] Danielsen M (2002). Characterization of the tetracycline resistance plasmid pMD5057 from *Lactobacillus plantarum* 5057 reveals a composite structure. Plasmid.

[53] Crowe-McAuliffe C, Murina V, Turnbull KJ, Huch S, Kasari M (2022). Structural basis for PoxtA-mediated resistance to phenicol and oxazolidinone antibiotics. Nat Commun.

[54] Cairns KA, Udy AA, Peel TN, Abbott IJ, Dooley MJ (2023). Therapeutics for vancomycin-resistant enterococcal bloodstream infections. Clin Microbiol Rev.

[55] Ausland C, Zheng J, Yi H, Yang B, Li T (2021). dbCAN-PUL: a database of experimentally characterized CAZyme gene clusters and their substrates. Nucleic Acids Res.

[56] Kiely LJ, Busca K, Lane JA, van Sinderen D, Hickey RM (2023). Molecular strategies for the utilisation of human milk oligosaccharides by infant gut-associated bacteria. FEMS Microbiol Rev.

[57] Gihawi A, Ge Y, Lu J, Puiu D, Xu A (2023). Major data analysis errors invalidate cancer microbiome findings. mBio.

[58] Roselli M, Finamore A, Britti MS, Konstantinov SR, Smidt H (2007). The novel porcine *Lactobacillus sobrius* strain protects intestinal cells from enterotoxigenic *Escherichia coli* K88 infection and prevents membrane barrier damage. J Nutr.

[59] Hynönen U, Kant R, Lähteinen T, Pietilä TE, Beganović J (2014). Functional characterization of probiotic surface layer protein-carrying Lactobacillus amylovorus strains. BMC Microbiol.

[60] Konstantinov SR, Smidt H, Akkermans ADL, Casini L, Trevisi P (2008). Feeding of *Lactobacillus sobrius* reduces *Escherichia coli* F4 levels in the gut and promotes growth of infected piglets. FEMS Microbiol Ecol.

[61] Tang Q, Yi H, Hong W, Wu Q, Yang X (2021). Comparative effects of *L. plantarum* CGMCC 1258 and *L. reuteri* LR1 on growth performance, antioxidant function, and intestinal immunity in weaned pigs. Front Vet Sci.

[62] Vela AI, García N, Latre MV, Casamayor A, Sánchez-Porro C (2007). *Aerococcus suis* sp. nov., isolated from clinical specimens from swine. Int J Syst Evol Microbiol.

[63] Park J, Friendship RM, Poljak Z, Weese JS, Dewey CE (2013). An investigation of exudative epidermitis (greasy pig disease) and antimicrobial resistance patterns of *Staphylococcus hyicus* and *Staphylococcus aureus* isolated from clinical cases. Can Vet J.

[64] Segura M, Fittipaldi N, Calzas C, Gottschalk M (2017). Critical *Streptococcus suis* virulence factors: are they all really critical?. Trends Microbiol.

[65] Uzal FA, Navarro MA, Asin J, Boix O, Ballarà-Rodriguez I (2023). Clostridial diarrheas in piglets: a review. Vet Microbiol.

[66] Sarowska J, Futoma-Koloch B, Jama-Kmiecik A, Frej-Madrzak M, Ksiazczyk M (2019). Virulence factors, prevalence and potential transmission of extraintestinal pathogenic *Escherichia coli* isolated from different sources: recent reports. Gut Pathog.

[67] Chen Z, Lv S, Zhang S, Yu Y, Ma J (2026). Identification of porcine-derived atypical intestinal pathogenic *Escherichia coli* reveals to a hidden threat of extraintestinal infection. Vet Microbiol.

[68] Camargo A, Ramírez JD, Kiu R, Hall LJ, Muñoz M (2024). Unveiling the pathogenic mechanisms of *Clostridium perfringens* toxins and virulence factors. Emerg Microbes Infect.

[69] Wu K, Yuan Y, Fang M, Liu Y, Yang D (2024). Genomic insights into cpb2-positive *Clostridium perfringens* and the potential biological function of cpb2 gene. *One Health Adv*.

[70] Abdullahi IN, Lozano C, Latorre-Fernández J, Zarazaga M, Stegger M (2025). Genomic analysis of multi-drug resistant coagulase-negative staphylococci from healthy humans and animals revealed unusual mechanisms of resistance and CRISPR-Cas system. Int Microbiol.

[71] Antonelli A, D’Andrea MM, Brenciani A, Galeotti CL, Morroni G (2018). Characterization of poxtA, a novel phenicol–oxazolidinone–tetracycline resistance gene from an MRSA of clinical origin. J Antimicrob Chemother.

[72] Zahedi Bialvaei A, Rahbar M, Yousefi M, Asgharzadeh M, Samadi Kafil H (2017). Linezolid: a promising option in the treatment of Gram-positives. J Antimicrob Chemother.

[73] Zhu Y, Yang W, Schwarz S, Xu Q, Yang Q (2022). Characterization of an MDR *Lactobacillus salivarius* isolate harbouring the phenicol-oxazolidinone-tetracycline resistance gene *poxtA*. J Antimicrob Chemother.

[74] Jiang Z, Yaqoob MU, Siddique A, Guang J, Ed-Dra A (2025). *Bergeyella zoohelcum*: a first case report of its association with respiratory diseases in swine in China. Vet Res Commun.

[75] Longhi G, Lugli GA, Tarracchini C, Fontana F, Bianchi MG (2024). From raw milk cheese to the gut: investigating the colonization strategies of *Bifidobacterium mongoliense*. Appl Environ Microbiol.

[76] Togo AH, Grine G, Khelaifia S, des Robert C, Brevaut V (2019). Culture of methanogenic archaea from human colostrum and milk. Sci Rep.

[77] Rattigan R, Lawlor PG, Cormican P, Crespo-Piazuelo D, Cullen J (2023). Maternal and/or post-weaning supplementation with *Bacillus altitudinis* spores modulates the microbial composition of colostrum, digesta and faeces in pigs. Sci Rep.

[78] de Oliveira IMF, Fredriksen S, Gutiérrez MF, Harmsen HJM, Boekhorst J (2025). Culturomics of the pig tonsil microbiome identifies new species and an untapped source of novel antimicrobials. Microbiome.

[79] Vlasblom AA, Duim B, Patel S, Luiken REC, Crespo-Piazuelo D (2024). The developing pig respiratory microbiome harbors strains antagonistic to common respiratory pathogens. mSystems.

[80] De La Cruz KF, Townsend EC, Alex Cheong JZ, Salamzade R, Liu A (2024). The porcine skin microbiome exhibits broad fungal antagonism. Fungal Genet Biol.

[81] Holman DB, Brunelle BW, Trachsel J, Allen HK (2017). Meta-analysis to define a core microbiota in the swine gut. mSystems.

[82] Oliveira IMF de, Ng DYK, van Baarlen P, Stegger M, Andersen PS (2022). Comparative genomics of *Rothia* species reveals diversity in novel biosynthetic gene clusters and ecological adaptation to different eukaryotic hosts and host niches. Microb Genom.

[83] Rumeau M, Chadi S, Pepke F, Beaumont M, Vicente CM (2025). Structure-dependent degradation of milk oligosaccharides by newly isolated intestinal commensal bacterial strains from suckling piglets and rabbits. BMC Microbiol.

[84] Lordan C, Roche AK, Delsing D, Nauta A, Groeneveld A (2024). Linking human milk oligosaccharide metabolism and early life gut microbiota: bifidobacteria and beyond. Microbiol Mol Biol Rev.

[85] Luis AS, Hansson GC (2023). Intestinal mucus and their glycans: a habitat for thriving microbiota. Cell Host Microbe.

[86] Chapman JA, Masi AC, Beck LC, Watson H, Young GR Human milk oligosaccharide metabolism by *Clostridium* species suppresses inflammation and pathogen growth. Microbiology.

[87] Chisoro P, Krogh U, Theil PK, Eskildsen M (2023). Characteristics of sows’ milk and piglet nutrient utilization during a 28-d lactation period. J Anim Sci.

[88] Bradshaw CV, Suarez Trujillo A, Luecke SM, Logan LD, Mohallem R (2021). Shotgun proteomics of homogenate milk reveals dynamic changes in protein abundances between colostrum, transitional, and mature milk of swine. J Anim Sci.

[89] Zhao H, Zhao S, Zhu Q, Chen J, Quan Z (2024). Label-free-based proteomic analysis reveals differential whey proteins of porcine milk during lactation. Food Chem X.

[90] Keel BN, Lindholm-Perry AK, Oliver WT, Wells JE, Jones SA (2021). Characterization and comparative analysis of transcriptional profiles of porcine colostrum and mature milk at different parities. BMC Genom Data.

[91] Dieplinger B, Egger M, Gabriel C, Poelz W, Morandell E (2013). Analytical characterization and clinical evaluation of an enzyme-linked immunosorbent assay for measurement of afamin in human plasma. Clin Chim Acta.

[92] Petruk G, Elvén M, Hartman E, Davoudi M, Schmidtchen A (2021). The role of full-length apoE in clearance of Gram-negative bacteria and their endotoxins. J Lipid Res.

[93] de Andrés J, Jiménez E, Chico-Calero I, Fresno M, Fernández L (2017). Physiological translocation of lactic acid bacteria during pregnancy contributes to the composition of the milk microbiota in mice. Nutrients.

