# Supplementary material for: Multi-omic characterization of the sow colostrum and milk microbiome and proteome
Source: Microb Genom. 2026 Jun 17;12(6):001726. doi: 10.1099/mgen.0.001726 (PMC13279891; doi:10.1099/mgen.0.001726)
Supplement: Supplementary Material 1. [file mgen-12-01726-s001.pdf]

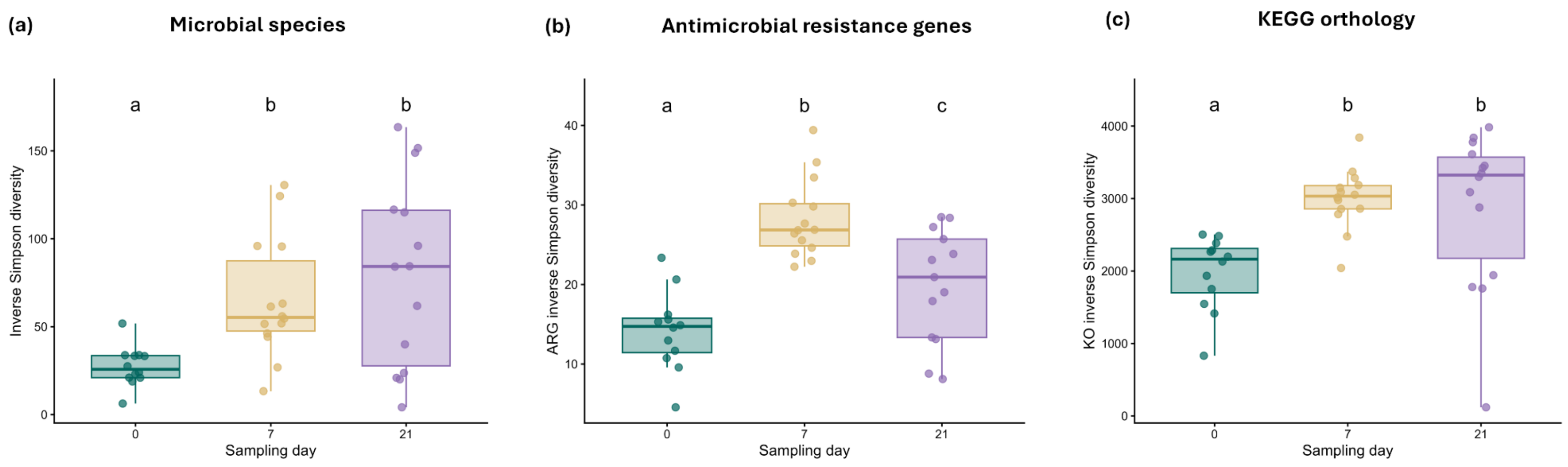

Figure S1. Box-and-whisker plots showing inverse Simpson diversity indices in colostrum (day 0) and milk samples (days 7 and 21) across sampling days for (a) microbial species, (b) antimicrobial resistance genes, and (c) KEGG orthologs. The centre line indicates the median, boxes represent the interquartile range, whiskers extend to 1.5 times the interquartile range, and points represent individual samples. Different lowercase letters indicate significant differences among sampling days within each panel.

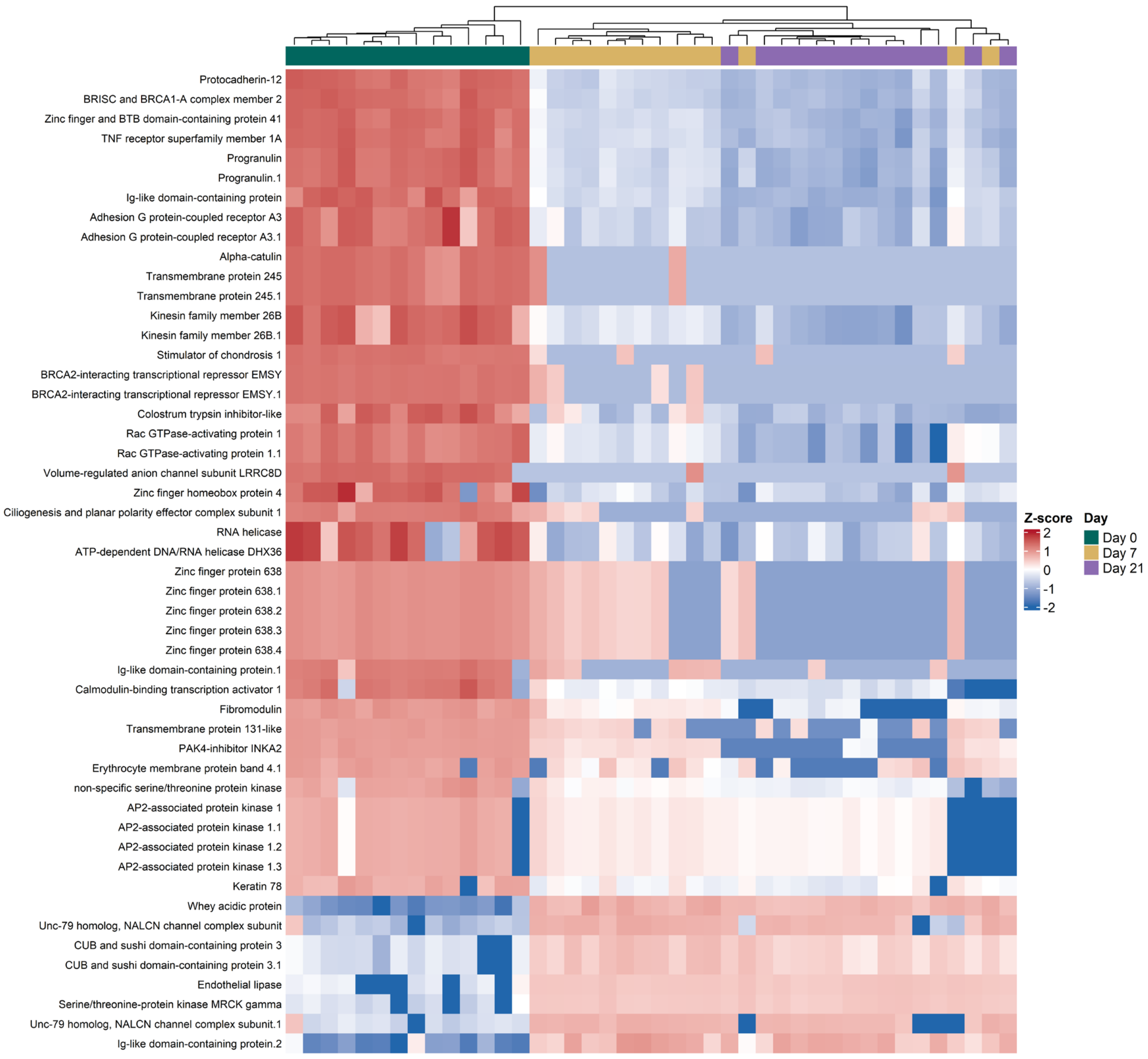

Figure S2. Heatmap of the 50 proteins with the largest absolute log2 fold changes identified in at least one of the three pairwise day comparisons. Protein abundances were log2-transformed and scaled by protein (row-wise z-score) to visualize relative temporal patterns across samples. Rows represent proteins and columns represent individual samples. Columns were clustered based on proteomic similarity, and the top annotation indicates sampling day. Red and blue indicate relatively higher and lower abundance, respectively, within each protein.
